# Pharmacologic Targeting of Histone H3K27 Acetylation/BRD4-dependent Induction of ALDH1A3 for Early-phase Drug Tolerance of Gastric Cancer

**DOI:** 10.1158/2767-9764.CRC-23-0639

**Published:** 2024-05-20

**Authors:** Jin Lee, Tetsuo Mashima, Naomi Kawata, Noriko Yamamoto, Shun Morino, Saori Inaba, Ayane Nakamura, Koshi Kumagai, Takeru Wakatsuki, Kengo Takeuchi, Kensei Yamaguchi, Hiroyuki Seimiya

**Affiliations:** 1 Division of Molecular Biotherapy, Cancer Chemotherapy Center, Japanese Foundation for Cancer Research, Tokyo, Japan.; 2 Department of Computational Biology and Medical Sciences, Graduate School of Frontier Sciences, The University of Tokyo, Tokyo, Japan.; 3 Gastroenterological Medicine, Cancer Institute Hospital, Japanese Foundation for Cancer Research, Tokyo, Japan.; 4 Division of Pathology, The Cancer Institute, Japanese Foundation for Cancer Research, Tokyo, Japan.; 5 Department of Life and Pharmaceutical Sciences, Graduate School of Pharmaceutical Sciences, Meiji Pharmaceutical University, Tokyo, Japan.; 6 Gastroenterological Surgery, Cancer Institute Hospital, Japanese Foundation for Cancer Research, Tokyo, Japan.; 7 Department of Upper Gastrointestinal Surgery, Kitasato University School of Medicine, Sagamihara, Kanagawa, Japan.

## Abstract

Anticancer drug-tolerant persister (DTP) cells at an early phase of chemotherapy reshape refractory tumors. Aldehyde dehydrogenase 1 family member A3 (ALDH1A3) is commonly upregulated by various anticancer drugs in gastric cancer patient-derived cells (PDC) and promotes tumor growth. However, the mechanism underlying the generation of ALDH1A3-positive DTP cells remains elusive. Here, we investigated the mechanism of ALDH1A3 expression and a combination therapy targeting gastric cancer DTP cells. We found that gastric cancer tissues treated with neoadjuvant chemotherapy showed high ALDH1A3 expression. Chromatin immunoprecipitation (ChIP)-PCR and ChIP sequencing analyses revealed that histone H3 lysine 27 acetylation was enriched in the *ALDH1A3* promoter in 5-fluorouracil (5-FU)-tolerant persister PDCs. By chemical library screening, we found that the bromodomain and extraterminal (BET) inhibitors OTX015/birabresib and I-BET-762/molibresib suppressed DTP-related ALDH1A3 expression and preferentially inhibited DTP cell growth. In DTP cells, BRD4, but not BRD2/3, was recruited to the *ALDH1A3* promoter and BRD4 knockdown decreased drug-induced ALDH1A3 upregulation. Combination therapy with 5-FU and OTX015 significantly suppressed *in vivo* tumor growth. These observations suggest that BET inhibitors are efficient DTP cell–targeting agents for gastric cancer treatment.

**Significance:**

Drug resistance hampers the cure of patients with cancer. To prevent stable drug resistance, DTP cancer cells are rational therapeutic targets that emerge during the early phase of chemotherapy. This study proposes that the epigenetic regulation by BET inhibitors may be a rational therapeutic strategy to eliminate DTP cells.

## Introduction

Gastric cancer is currently the fourth most common cause of mortality among various types of cancers (1). Conventional chemotherapy based on 5-fluorouracil (5-FU) and other cytotoxic drugs, molecular targeted drugs, and immune checkpoint inhibitors is used as standard pharmacologic therapeutics for the treatment of patients with metastatic, recurrent, or advanced gastric cancer (2). However, their therapeutic efficacy is largely limited by intrinsic and acquired drug resistance, and early relapse after treatment.

Tumor tissues contain heterogeneous cancer cells (3). In particular, drug-resistant cancer cells remain as drug-tolerant persister (DTP) cells after drug treatment, which hinders the efficacy of pharmacologic treatments (4). To improve the therapeutic outcomes, DTP cells should be eradicated in the early phase of drug treatment to prevent expansion of cancer cells that acquire stable drug resistance. Recent studies have focused on the maintenance of DTP cancer cells after drug treatment (5–9). Although the mechanisms vary depending on the cancer cell type or therapeutic agent, observations suggest that DTP cells are residual drug-resistant cancer cells that exist in tumor tissues or are phenotypically induced after drug exposure (10).

Epigenetic regulation, which is closely linked to chromatin modification, plays an essential role in the regulation of cancer cell plasticity and malignant phenotypes (11, 12). Histone modifications contribute to dramatic changes in cellular contexts through global transcriptional reprogramming and are triggered by intrinsic and external stimuli (13, 14). Active histone marks, such as histone H3 lysine 4 trimethylation (H3K4me3) and lysine 27 acetylation (H3K27ac), and repressive marks, such as H3K9me3 and H3K27me3, cause transcriptional activation and repression, respectively. Histone marks are also recognized by other regulators, including reader bromodomain and extraterminal (BET) domain family proteins. Although several external stimuli have been proposed to induce histone modification changes, such as oxidative stress and DNA damage, the detailed mechanisms associated with DTP cell regulation remain elusive.

Aldehyde dehydrogenase 1 family, member A3 (ALDH1A3) is one of 19 ALDH isoforms that convert aldehydes to carboxylates. ALDH1A3 is overexpressed in various cancers and is a marker of cancer stem cells, which contributes to cancer initiation, metastasis, therapeutic resistance, and relapse (15–17). ALDH1A3 mediates diverse intracellular events, such as retinoic acid biosynthesis and mitochondrial metabolism (18). Our previous study showed that ALDH1A3 expression is highly upregulated by treatment with cytotoxic anticancer drugs such as 5-FU, 7-ethyl-10-hydroxycamptothecin (also known as SN-38), cisplatin (CDDP), and paclitaxel in gastric cancer patient-derived cells (PDC) and is involved in cancer cell survival (19). However, it remains unclear how ALDH1A3-overexpressing DTP cells persist after drug treatment, and which types of signals contribute to DTP cell maintenance.

In this study, we demonstrated genome-wide histone modifications in gastric cancer DTP cells and the involvement of epigenetic machinery in DTP cell maintenance after chemotherapy. Our findings provide a novel molecular basis for the development of a therapeutic approach for eradicating gastric cancer DTP cells by targeting epigenetic regulators.

## Materials and Methods

### IHC

Formalin-fixed, paraffin-embedded (FFPE) tissues of 40 gastric cancers, including 20 patients treated with neoadjuvant chemotherapy (NAC) with fluoropyrimidines and platinum agents and 20 treatment-naïve controls, were obtained from the Cancer Institute Hospital, Japanese Foundation for Cancer Research (JFCR) from 2017 to 2019. The tissues were histologically evaluated under approval from the Institutional Review Board of JFCR with written informed consent of patients in accordance with Declaration of Helsinki. FFPE sections were deparaffinized by soaking in xylene. To replace xylene with ethanol, the sections were immersed in 70% ethanol and distilled water (DW). Using a 1:10-diluted DAKO REAL Target Retrieval Solution (Dako), antigen activation was conducted for 30 minutes. After washing with DW, sections were immersed in 0.3% hydrogen peroxide in methanol for 10 minutes at room temperature. The sections were washed with TBS and 0.1% Tween-20 (Nacalai Tesque) and blocked for 10 minutes at room temperature with Blocking One Histo (Nacalai Tesque). Each section was incubated overnight with primary antibodies in TBST at 4°C. After washing with TBST, the sections were stained with EnVision+ Dual Link System-HRP (Dako) for 30 minutes at room temperature. For color development, the sections were treated with the Liquid DAB+ substrate-chromogen system (Dako) for 1 minute at room temperature. After washing with water, the sections were stained with hematoxylin for 10 seconds and washed with DW. The sections were dehydrated with 70% ethanol, 100% ethanol, and then xylene. Each section was sealed with Mount-quick (Daido Sangyo). ALDH1A3 expression was evaluated by a board-certified pathologist (N. Yamamoto) with more than 20 years of experience in gastrointestinal pathology. The expression intensity was scored using a 4-tier system: 0, no reactivity; 1, faint reactivity; 2, moderate reactivity; 3, strong reactivity. The percentage of cancer cells with positive reactivity at any intensity was recorded. H-score is calculated on the basis of the following formula: H-score = expression intensity × percentage of signal-positive cancer cells/100. IHC analysis was performed as described in Supplementary Materials and Methods.

### Cell Culture

Gastric cancer PDC lines JSC15-3 and JSC18-1 were established in JFCR from 2015 to 2018 under approval from the Institutional Review Board of JFCR, with written informed consent from patients in accordance with Declaration of Helsinki, as described previously (20). JSC15-3 cells were cultured in ACL4/F12 (1:1) medium (Nacalai Tesque) supplemented with 5% heat-inactivated FBS and 100 µg/mL kanamycin (Meiji Seika Pharma). JSC18-1 cells were cultured in ACL4/RPMI (1:1) medium supplemented with 5% heat-inactivated FBS and 100 µg/mL kanamycin. Cells were routinely tested for *Mycoplasma* contamination by PCR using the primers 5′-CACCATCTGTCACTCTGTTAACC-3′ and 5′-GGAGCAAACAGGATTAGATACCC-3′. *Mycoplasma* testing was also performed in 2021 by ICLAS monitoring center, Central Institute for Experimental Animals (Kanagawa, Japan).

### Vector Construction and Establishment of Genetically Engineered Cells

To establish ALDH1A3-overexpressing cells, the human *ALDH1A3* open reading frame sequence was amplified by PCR using cDNA from JSC15-3 cells as a template, and then cloned into a pLPCX retrovirus vector (Takara, RRID:Addgene_44471) using a DNA ligation kit Ver 2.1 (Takara). The primers used for amplification were as follows: *ALDH1A3* forward primer, 5′-TTTTGAATTCAGGAGCCATGGCCACCGCTAAC-3′ and *ALDH1A3* reverse primer, 5′-TTTTATCGATCTTTCCTTCAGGGGTTCTTGTC-3′. Retroviruses expressing the ALDH1A3 gene and control viruses (mock) were produced by transfecting GP2-293 cells (RRID:CVCL_WI48) with pLPCX-ALDH1A3 and pLPCX (control) together with the pVSV-G packaging vector, (RRID:Addgene_138479), respectively. JSC15-3 cells were infected with these viruses as described previously (21). Infected cells were selected using medium containing 1 µg/mL puromycin. Stable ALDH1A3 overexpression was confirmed by Western blot analysis and qRT-PCR.

We knocked in the internal ribosome entry site (IRES)-*green fluorescent protein* (*gfp*) gene into the *ALDH1A3* 3′-untranslated region (UTR) of JSC15-3 cells by CRISPR/Cas9-mediated genome editing and constructed a donor vector. The 5′ and 3′ homology arms, HR1 (915 bp) and HR2 (774 bp), respectively, around the *ALDH1A3* 3′-UTR were amplified by PCR and then cloned into the HR180PA-1 vector (SBI) containing the IRES-*gfp* sequence and EF-1 promoter-driven puromycin-resistant gene between two HR insertion sites using an In-Fusion HD cloning kit (Takara; ref. 22). The primers used for amplification were as follows: HR1 forward primer, 5′-GTGGCCACCTCTACCTTCATCT-3′ and HR1 reverse primer, 5′-TACCGAGCTCGAATTCTTTCCTTCAGGGGTTCTTGTCG-3′; HR2 forward primer, 5′-AACCTAGATCGGATCCGGCGGAATGTGGCAGAT-3′ and HR2 reverse primer, 5′-TGCTGTTGTGGCGTTAGAAGAT-3′. The knock-in reaction was performed by co-transfection of the donor vector with crRNA targeting the *ALDH1A3* 3′-UTR, tracrRNA (Horizon Discovery), and Cas9 plasmid (Horizon Discovery) using Dharmafect Duo (Horizon Discovery), followed by selection with 1 µg/mL puromycin (crRNA target sequence: 5′-GAACCCCTGAAGGAAAGGCG-3′). After cloning the knock-in cells, we validated the knock-in of the IRES-*gfp* sequence in the *ALDH1A3* 3′-UTR by genomic PCR around the target site and sequencing the amplified PCR products. The primers used for amplification were as follows: forward primer #1, 5′-GGACCACACTTTGAGAACCA-3′ and reverse primer #1,5′-TGGTTCCTCTGAGTTTCACC-3′; forward primer #2, 5′-CCTGATATCAAACATATAACTTCG-3′ and reverse primer #2: 5′-GTGAACGTGATAGAAATGCG-3′. We also confirmed dependency of GFP expression on ALDH1A3 expression by ALDH1A3 knockdown using specific siRNAs.

### Western Blot Analysis

Cells were lysed for 30 minutes on ice with a lysis buffer consisting of 50 mmol/L Tris-HCl (pH 8.0), 150 mmol/L NaCl, 1% Nonidet P-40 (NP-40; Nacalai Tesque), 2% protease inhibitors (Nacalai Tesque), and 150 mmol/L dithiothreitol. The cell lysates were centrifuged at 1,600 × *g* for 10 minutes at 4°C. Supernatants were collected as whole-cell lysates. Western blot analysis was performed as described in the Supplementary Materials and Methods.

### qRT-PCR

Total RNA was extracted using the RNeasy Mini Kit (Qiagen). cDNA was synthesized using the ReverTra Ace qPCR RT Master Mix (Toyobo). qRT-PCR was performed using Power SYBR Green PCR Master Mix (Applied Biosystems) in Step One Plus (Thermo Fisher Scientific). Primers used are listed in Supplementary Table S1. GAPDH was used as an internal control to normalize data.

### Mouse Xenograft Model

All animal procedures were performed using protocols approved by the JFCR Animal Care and Use Committee. To evaluate the effect of ALDH1A3 overexpression on tumor growth, JSC15-3/ALDH1A3 [overexpression (O/E)] or JSC15-3/mock cells (2 × 10^3^ cells/site) were suspended in 500 µL Hank's Balanced Salt Solution with 500 µL Matrigel (Corning) and subcutaneously implanted into 5-week-old female NOD-SCID mice (The Jackson Laboratories; *n* = 6 per group). On day 66 after implantation, tumor sizes [length (*L*) and width (*W*)] were measured using a digital caliper to calculate the tumor volume as (*L* × *W*^2^)/2. Details for evaluation of the therapeutic effect of 5-FU and OTX015 are described in Supplementary Materials and Methods. We also measured the mouse body weight to estimate adverse effects of the treatment.

### Chemical Compounds

The SCADS inhibitor kit, a chemical compound library of compounds, including cell signaling pathway inhibitors, molecular targeted drugs, and conventional anticancer drugs (Supplementary Table S2), was provided by the Molecular Profiling Committee, Grant-in-Aid for Scientific Research on Innovative Areas “Platform of Advanced Animal Model Support (AdAMS)” from The Ministry of Education, Culture, Sports, Science and Technology, Japan (KAKENHI 16H06276). Details of other chemical compounds are described in Supplementary Materials and Methods.

### Cell Proliferation Assay

To evaluate cell proliferation, an MTT [3-(4,5-dimethyl-2-thiazolyl)-2,5-diphenyltetrazolium bromide] assay was performed as described in Supplementary Materials and Methods.

### Time-lapse Imaging

Cells (5 × 10^4^) were seeded in a 12-well plate and treated with 5-FU and DMSO. Cell behavior was then monitored with a BioStation CT system (Nikon) under the following conditions: picture position of tiling, 3 × 3; × 10 magnification, ch2 (Ex/Em 438/483), 800 ms of exposure time and 240 luminance; scheduling 0, 12, 36, and 60 hours. Data trimming was performed with CL-Quant software (Nikon).

### Flow Cytometry

Cells were treated with DMSO and test compounds at the IC_50_ value of the parental cells. Then, the cells were collected in microtubes, centrifuged, and resuspended with FACS buffer consisting of 10% FBS, 1 mol/L HEPES (pH 7), 2 mmol/L ethylenediamine tetraacetic acid (EDTA), and PBS. Flow cytometry was performed using FACSLyric and FACSmelody (BD Biosciences).

### Chromatin Immunoprecipitation (ChIP)-PCR and ChIP Sequencing Analyses

JSC15-3 cells were treated with 3 µmol/L 5-FU or DMSO as controls. Cells (2 × 10^7^) were fixed with 1% formaldehyde for 10 minutes. Then, 10 × glycine solution was added to the cells, followed by incubation for 5 minutes at room temperature. After washing with cold PBS, the cells were collected using PBS containing a 200 × dilution of a protein inhibitor cocktail. The cells were subjected to chromatin immunoprecipitation (ChIP) using a SimpleChIP Enzymatic Chromatin IP Kit (Magnetic Beads; Cell Signaling Technology) in accordance with the manufacturer's instructions. Detailed procedures for ChIP-PCR and chromatin immunoprecipitation sequencing (ChIP-seq) data analysis are described in Supplementary Materials and Methods.

### RNA-sequencing Data Analysis

JSC15-3 cells were treated with 3 µmol/L 5-FU or DMSO as the control for 5 days. RNAs were extracted using an RNeasy Mini kit. The quality of RNA samples was confirmed by Agilent 2100 Bioanalyzer (Agilent). RNA sequencing (RNA-seq) was performed at Macrogen and GeneBay, using standard protocols. The quality of the raw FASTQ files was evaluated using FastQC (version 0.11.9). The adapters were removed using Trimmomatic (version 0.39). The reads were aligned to human hg38 and gene expression was quantified using FeatureCounts of the Subread package (version 2.0.1). After filtering low-expression genes and normalization, upregulated and downregulated genes were extracted using edgeR (version 3.40.0). A heat map was generated using ggplot2 (version 3.4.3).

### Drug Screening

ALDH1A3-GFP cells were seeded at 1,600 cells/well in a 96-well plate and treated with the compounds in the SCADS inhibitor kit at three concentrations (0.16, 0.8, and 4 µmol/L) and various epigenetic chemical compounds at two concentrations (1 and 3 µmol/L) for 5 days. The number of cells and GFP (ALDH1A3) expression were quantitated using an Operetta CLS (PerkinElmer). The relative cell number and GFP expression were calculated as the ratio of each value to that of the three averaged replicates of the DMSO-treated cells.

### siRNA and Antibodies

The siRNAs and antibodies used in this study are described in Supplementary Materials and Methods.

### Statistical Analysis

Experiments were performed with at least three independent biological replicates. Data are presented as mean ± SD. Statistical significance was determined using one-way ANOVA, two-tailed *t* test, or one-tailed Mann–Whitney rank-sum test. Except for RNA-seq and ChIP-seq data, statistical and correlation analyses were conducted using GraphPad Prism 9.0 software (GraphPad, RRID:SCR_002798). Statistical significance of the sequencing data was determined using EdgeR (version 3.40.0) in R studio.

### Data Availability

Sequencing data were deposited in the NCBI Gene Expression Omnibus (GEO, RRID:SCR_005012) under accession number GSE245889. Other data supporting the findings of this study are available from the corresponding author upon reasonable request.

### Code Availability

Custom code was not used in this study. RNA-seq data were analyzed using FastQC (version 0.11.9, RRID:SCR_014583), Trimmomatic (version 0.39, RRID:SCR_011848), STAR (version 2.7.9a, RRID:SCR_004463), FeatureCount (RRID:SCR_012919)/Subread (version 2.0.1), EdgeR (version 3.40.0, RRID:SCR_012802), and ggplot2 (version 3.4.3, RRID:SCR_014601). ChIP-seq data were analyzed using bwa mem (version 0.7.17), samtools (version 1.15.1, RRID:SCR_002105), MACS3 (version 3.0.0b3), bedtools (version 2.27.1), deeptools (version 3.5.1), IGV (version 2.16.0), ClusterProfiler (RRID:SCR_016884), ChIPpeakAnno (version 3.32.0, RRID:SCR_012828), ChIPseeker (version 1.34.1), DiffBind (version 3.8.4, RRID:SCR_012918), and EdgeR (version 3.40.0), csaw (version 1.32.0) in R packages (4.2.1).

## Results

### Enhanced Expression of ALDH1A3 in Gastric Cancer Treated with NAC

**FIGURE 1:**
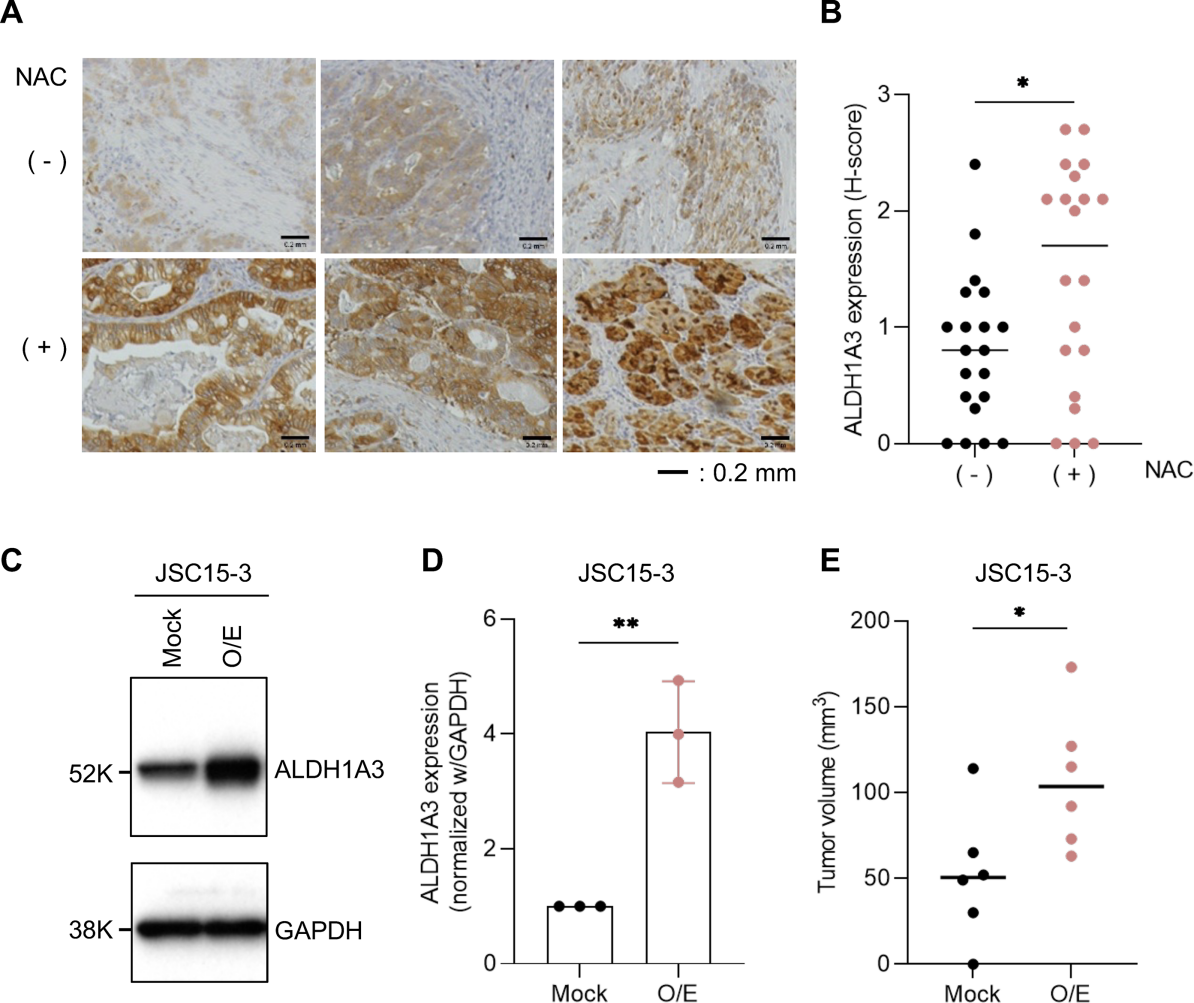
ALDH1A3 overexpression in clinical gastric cancer treated by NAC and its implication for tumor growth. **A,** IHC of ALDH1A3 in FFPE tissues of 40 gastric cancer cases. Twenty cases treated by NAC with fluoropyrimidines and platinum agents, and 20 treatment-naïve controls were analyzed. Representative images of three NAC (−) and three NAC (+) specimens were shown. **B,** Quantitation of ALDH1A3 expression (H-score) in A. Details for the calculation are described in Materials and Methods. Western blot (**C**) and qRT-PCR (**D**) analyses of ALDH1A3 expression in mock and ALDH1A3-overexpressing (O/E) gastric cancer patient-derived JSC15-3 cells. In D, each column represents the mean ± SD of three technical replicates. Data are representative of at least three independent experiments. **E,** Effect of ALDH1A3 overexpression on xenografted tumor growth. NOD-SCID mice were subcutaneously implanted with mock and O/E JSC15-3 cells. At day 66 after implantation, tumor volumes were measured to evaluate the statistical significance of the difference between the groups. *, *P* < 0.05; **, *P* < 0.01, determined by the two-tailed *t* test.

We previously demonstrated that ALDH1A3 expression is commonly upregulated by anticancer drugs in multiple gastric cancer PDC lines and mouse xenograft models (19). To evaluate the effects of anticancer drugs on ALDH1A3 expression in a clinical context, we analyzed ALDH1A3 expression in FFPE tissues from 40 gastric cancers, including 20 cases treated with NAC with fluoropyrimidines and platinum agents and 20 treatment-naïve cases. As shown in Fig. 1A and B, tumor tissues from patients treated with NAC showed significantly higher ALDH1A3 expression than those from treatment-naïve patients. To examine the effect of ALDH1A3 upregulation on cancer growth *in vivo*, we exogenously overexpressed ALDH1A3 in the gastric cancer PDC line JSC15-3 (Fig. 1C and D) and subcutaneously implanted it into NOD-SCID mice. ALDH1A3 O/E significantly promoted tumor growth *in vivo* compared with that in mock cells (Fig. 1E). Moreover, we examined the 5-FU sensitivity of the mock and exogenous ALDH1A3-O/E gastric cancer PDCs. As a result, ALDH1A3 O/E conferred marginal but statistically significant resistance to 5-FU (GI_50_ of ALDH1A3 O/E cells and mock cells were 22.7 and 6.6 µmol/L, respectively; Supplementary Fig. S1A). On the other hand, ALDH1A3 O/E did not cause CDDP resistance, which suggests differential involvement of ALDH1A3 in the resistance to these agents (Supplementary Fig. S1B). These observations suggest that ALDH1A3 upregulation by chemotherapy in clinical gastric cancer has a positive effect on the growth and survival of residual tumors.

### Anticancer Drugs Induce ALDH1A3-overexpressing DTP Cells

**FIGURE 2:**
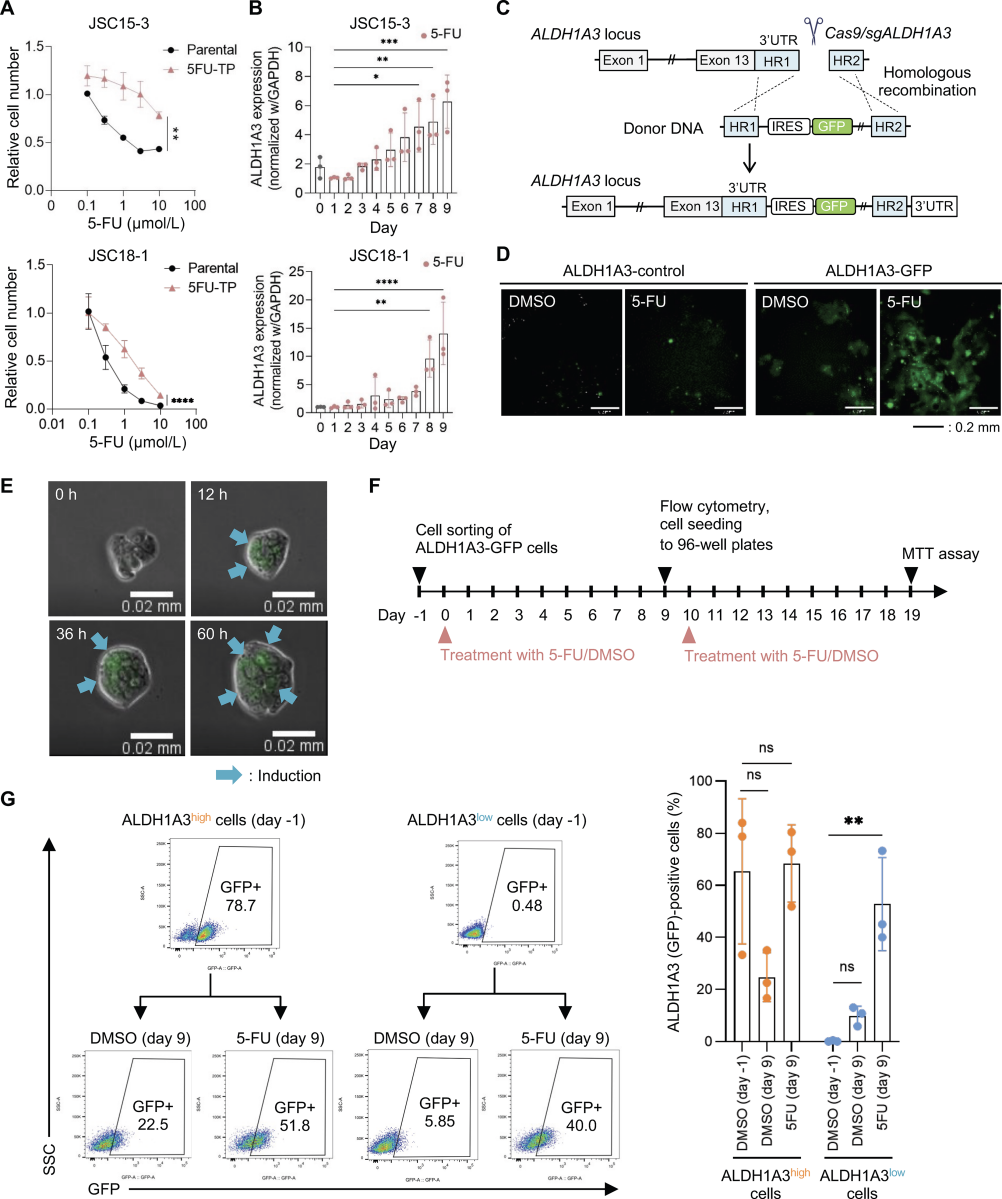
Induction of ALDH1A3-overexpressing DTP cells upon treatment with 5-FU. **A,** Sensitivity of 5FU-TP cells to 5-FU. Parental JSC15-3 (top) and JSC18-1 (bottom) cells and their 5FU-TP cells were treated with 5-FU at the indicated concentrations for 9 days. Data are representative of at least three independent experiments. **, *P* < 0.01; ****, *P* < 0.0001, two-tailed *t* test. **B,** ALDH1A3 induction by 5-FU. Cells were treated with 3 µmol/L 5-FU or DMSO and then subjected to qRT-PCR. Data are representative of at least three independent experiments. *, *P* < 0.05; **, *P* < 0.01; ***, *P* < 0.001; ****, *P* < 0.0001, one-way ANOVA. **C,** Experimental design of *gfp* knock-in into the *ALDH1A3* locus using CRISPR/Cas9-mediated genome editing. IRES, internal ribosome entry site. In construct schemes, only representative genetic elements are shown. **D,** Visualization of ALDH1A3-GFP expression. Cells were treated with 3 µmol/L 5-FU or DMSO for 5 days and then analyzed by fluorescence microscopy. **E,** Live imaging of ALDH1A3-GFP cells. Cells were treated with 3 µmol/L 5-FU or DMSO for 60 hours. Images were obtained at 0, 12, 36, 60 hours. Blue arrows indicate ALDH1A3 (GFP) induction. **F,** Schema for experimental schedule for cell sorting with flow cytometry. **G,** FACS of ALDH1A3-GFP expression. ALDH1A3-GFP cells were divided into ALDH1A3^high^ and ALDH1A3^low^ cells (day −1). Each cell fraction (day 0) was treated with 3 µmol/L 5-FU or DMSO for 9 days and then subjected to flow cytometry (day 9). Right bar graph shows a quantitative representation of triplicate data. **, *P* < 0.01; ns, not significant, one-way ANOVA.

To confirm drug tolerance and ALDH1A3 expression in residual gastric cancer cells after anticancer drug treatment, we collected DTP cells after treating the gastric cancer PDC lines JSC15-3 and JSC18-1 with 5-FU or CDDP for 9 days and compared their drug sensitivities with those of the parental cells. The 5FU–tolerant persister (5FU-TP) and CDDP-tolerant persister (CDDP-TP) cells showed resistance to each drug compared with the parental cells (Fig. 2A; Supplementary Fig. S1C). We also showed that the cells treated with 5-FU for 5 days are also resistant to 5-FU (Supplementary Fig. S1D). We further confirmed that the growth of the cells treated with 5-FU for 5 days was significantly slowed down (Supplementary Fig. S1E). To assess ALDH1A3 amplification after the drug treatment, we performed qPCR analysis with the genomic DNAs of DMSO and 5-FU treated cells. As a result, no significant ALDH1A3 genomic amplification was observed (Supplementary Fig. S1F). ALDH1A3 transcripts were time-dependently increased in both PDC lines after 5-FU treatment (Fig. 2B). This ALDH1A3 upregulation was significantly suppressed again after the removal of 5-FU for 2 weeks (Supplementary Fig. S1G), suggesting a reversible phenotype. We evaluated the mRNA levels of typical cancer stem markers, CD44, LGR5, SOX2, and TROY, using qRT-PCR. While CD44 and SOX2 were upregulated in JSC15-3 and JSC18-1 DTP (5FU-TP) cells, respectively, other markers were not upregulated in those cells (Supplementary Fig. S1H). We further evaluated invasive potential of 5FU-TP cells. As shown in Supplementary Fig. S1I, the DTP cells did not show any increased invasive potential. These results suggest that the persister phenotype is not related to typical cancer stemness. In our previous study, treatment-naïve gastric cancer PDCs included a certain fraction of ALDH1A3^high^ cells even before drug treatment (19). These observations suggest two possible mechanisms for ALDH1A3-overexpressing DTP cell accumulation after anticancer drug treatment. Specifically, ALDH1A3^high^ cancer cells that existed before drug treatment were selected after drug exposure or drug treatment induced ALDH1A3-overexpressing cells.

To evaluate these possibilities, we knocked in the IRES-*gfp* gene into the *ALDH1A3* 3′-UTR of JSC15-3 cells using CRISPR/Cas9-mediated genome editing, which enabled us to monitor ALDH1A3 expression by tracing the co-expressed GFP signal in live cells (Fig. 2C; Supplementary Fig. S2A and S2B). In knock-in cells (ALDH1A3-GFP), GFP was induced by 5-FU at the mRNA and protein levels (Supplementary Fig. S2C and S2D). Flow cytometry detected GFP fluorescence in knock-in cells but not in parental cells (Fig. 2D; Supplementary Fig. S2E). The number of GFP-positive cells was markedly increased by 5-FU in a time- and dose-dependent manner (Supplementary Fig. S2F and S2G). In knock-in cells, 5-FU and CDDP induced GFP expression (Supplementary Fig. S2H). Using a live imaging system, we found that GFP-positive cells were induced by 5-FU treatment, even from GFP-negative cells (Fig. 2E).

To clarify the generation step of ALDH1A3-overexpressing persister cells, we sorted ALDH1A3(GFP)^high^ and ALDH1A3(GFP)^low^ cells from knock-in cells and monitored GFP expression levels after 5-FU treatment (Fig. 2F). As a result, preexisting ALDH1A3^high^ cells were unstable and were maintained only in the presence of 5-FU. However, approximately 50% of the ALDH1A3^low^ cells were converted to ALDH1A3^high^ cells after 5-FU treatment (Fig. 2G). ALDH1A3^high^- and ALDH1A3^low^-derived 5FU-TP cells exhibited 5-FU resistance, although the latter did not show statistical significance (Supplementary Fig. S2I). Meanwhile, CDDP-TP cells from ALDH1A3^high^ and ALDH1A3^low^ cells were comparably resistant to CDDP as compared with DMSO-treated control cells (Supplementary Fig. S2J). We further compared 5-FU sensitivity of 5FU-TP cells and CDDP sensitivity of CDDP-TP cells from ALDH1A3^high^ and ALDH1A3^low^ cells, respectively. As shown in Supplementary Fig. S2K, both 5FU-TP and CDDP-TP cells from ALDH1A3^high^ and ALDH1A3^low^ cells exhibited comparable sensitivities to 5-FU and CDDP, respectively. These results indicate that ALDH1A3^high^ cells are mainly generated by 5-FU or CDDP rather than selection of preexisting ALDH1A3^high^ cells.

### Epigenetic Regulation of ALDH1A3 and Persister Gene Expression in Gastric Cancer DTP Cells

**FIGURE 3:**
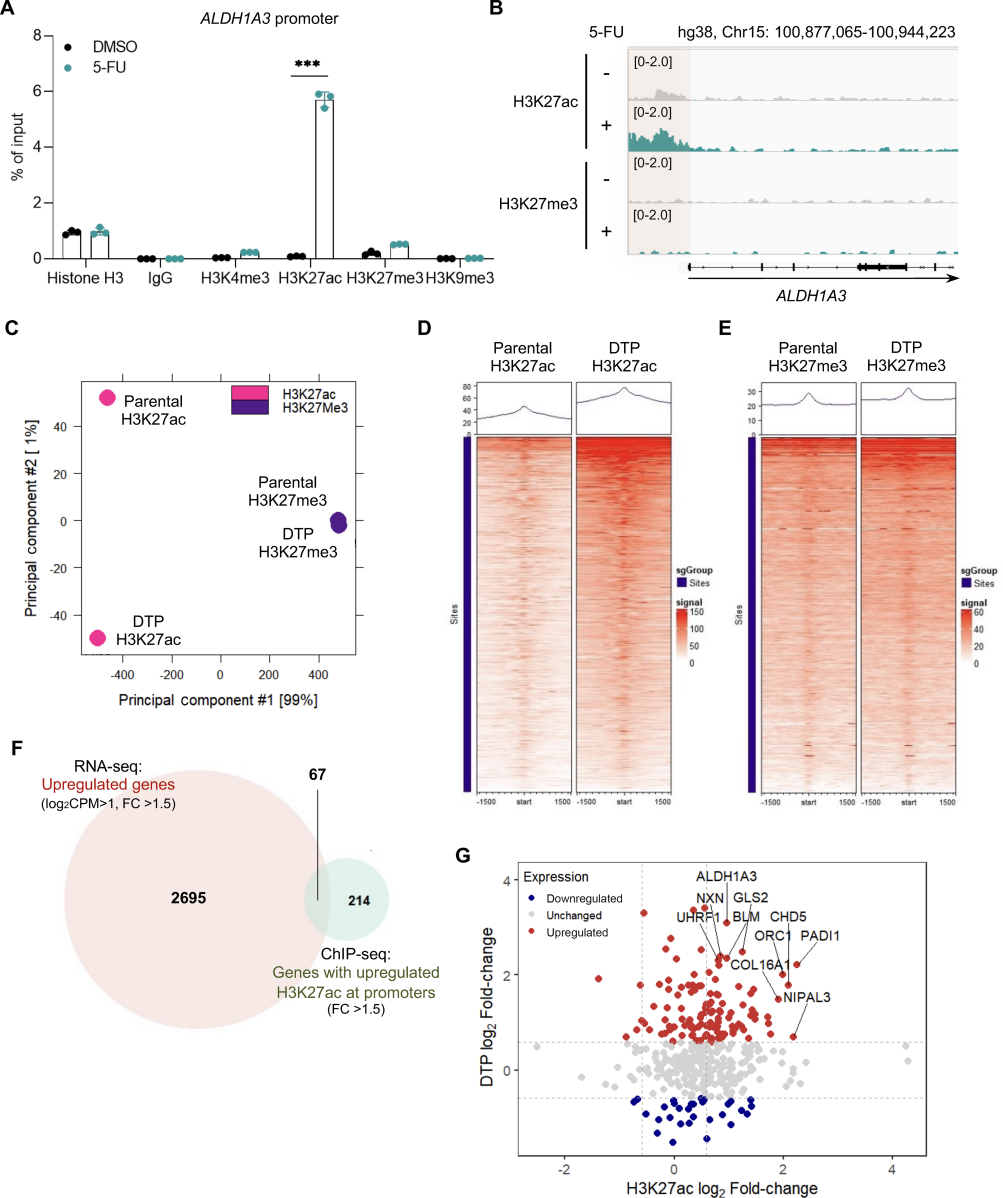
Histone H3 lysine 27 acetylation marks ALDH1A3 and other DTP genes. **A,** ChIP-PCR analysis of histone modification in DTP cells in the *ALDH1A3* promoter region. JSC15-3 cells were treated with 3 µmol/L 5-FU or DMSO for 5 days. Each bar represents the mean ± SD of three technical replicates. Experiments were performed at least three times, and representative data are shown. ***, *P* < 0.001, two-tailed *t* test. **B,** ChIP-seq analysis of histone modification in DTP cells in the *ALDH1A3* promoter region. Cells were treated as described in A. Genome browser view of chromatin accessibility enrichment of H3K27ac and H3K27me3 at the *ALDH1A3* locus is shown. **C,** Principal component analysis plot of H3K27ac and H3K27me3 peaks in parental and 5FU-TP (DTP) cells. Default profile plots of H3K27ac (**D**) and H3K27me3 (**E**) drawn using DiffBind package in R (version 3.8.4). Each sample was normalized to the input. **F,** Venn diagram of upregulated genes and H3K27ac peaks in DTP cells. Upregulated genes in DTP cells were identified by RNA-seq analysis of JSC15-3 cells treated with 3 µmol/L 5-FU and DMSO for 5 days. Genes with a read count of more than 10 (CPM > 2) and fold change (FC) of greater than 1.5 were extracted using EdgeR and csaw packages in R, among which 67 genes overlapped with genes exhibiting enhanced H3K27ac signals in DTP cells as determined by ChIP-seq analysis. **G,** Top 10 genes with upregulated expression and H3K27ac levels in DTP cells.

To identify the regulators of persister cell maintenance related to ALDH1A3 induction, we performed ChIP-PCR for four major histone modifications (H3K4me3, H3K27ac, H3K27me3, and H3K9me3) in the *ALDH1A3* promoter. As shown in Fig. 3A, H3K27ac levels were elevated in the *ALDH1A3* promoter of 5FU-TP cells. Conversely, no marked differences were observed in H3K4me3, H3K27me3, or H3K9me3 levels at the *ALDH1A3* promoter in parental and 5FU-TP cells. To evaluate global changes in the chromatin state in DTP cells, we performed ChIP-seq analyses. Again, H3K27ac, but not H3K27me3, increased in the *ALDH1A3* promoter region of DTP cells (Fig. 3B). Principal component analysis further revealed that genome-wide alterations of H3K27ac, but not H3K27me3, occurred in DTP cells compared with parental JSC15-3 cells (Fig. 3C). The levels of H3K27ac peaks, which were mainly located in the promoter regions but not global regions (Supplementary Fig. S3A–S3C), were elevated around transcription start sites in DTP cells (Fig. 3D). Conversely, H3K27me3 levels were comparable between parental and DTP cells (Fig. 3E). These observations suggest that the accumulation of H3K27ac in DTP cells specifically occurs in persister gene promoter regions. We extracted 2,762 genes that were upregulated by more than 1.5-fold [counts per million (CPM) >2] in DTP cells from the RNA-seq data (Fig 3F). Next, we performed differential expression analysis using parental and DTP H3K27ac peaks. Among 281 genes that exhibited increased H3K27ac levels by more than 1.5-fold at the promoter regions in DTP cells, 67 genes were overlapped with the upregulated genes in DTP cells. Gene ontology (GO) analysis revealed that the upregulated genes in DTP cells were related to the regulation of the cell cycle process and DNA replication (Supplementary Fig. S3D). Genes with enhanced H3K27ac marks in DTP cells were associated with regulation of the mitotic cell cycle. The 67 overlapping genes were related to the regulation of the mitotic cell cycle and retinoid metabolic processes (Supplementary Fig. S3D). Consistent with these GO terms, JSC15-3 cells were arrested in the S-phase of the cell cycle after 5-FU treatment (Supplementary Fig S3E; ref. 23). Notably, among the 67 genes, ALDH1A3 was highly upregulated with enhanced H3K27ac marks in DTP cells (Fig. 3G). These observations suggest that the induction of ALDH1A3 and other DTP-related genes is regulated by histone acetylation on their promoters.

### BET Inhibitors Suppress ALDH1A3 Expression in Gastric Cancer DTP Cells

**FIGURE 4:**
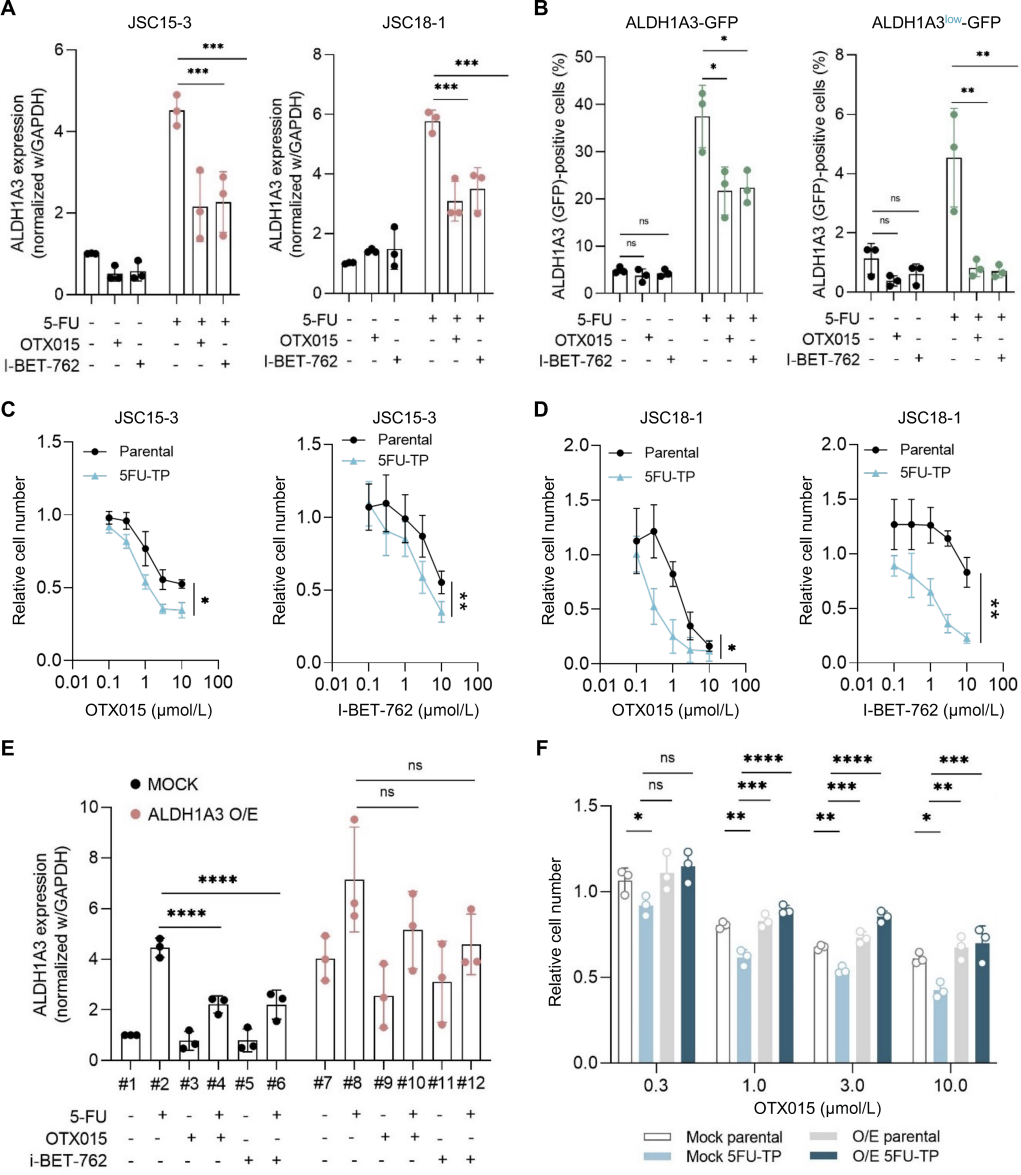
BET inhibitors suppress ALDH1A3 expression and growth of DTP cells. **A,** Effects of BET inhibitors on 5-FU–induced mRNA expression of ALDH1A3 in JSC15-3 (left) and JSC18-1 (right) cells. Cells were treated with 3 µmol/L 5-FU in the presence or absence of 1 µmol/L OTX015 or 3 µmol/L I-BET-762 for 5 days. Then, RNAs were prepared and subjected to qRT-PCR. **B,** FACS analysis of ALDH1A3-GFP (left) and ALDH1A3^low^-GFP (right) JSC15–3 cells. Cells were treated as described in A and subjected to flow cytometry. In A and B, experiments were conducted with three technical replicates and were repeated at least three times. *, *P* < 0.05; **, *P* < 0.01; ***, *P* < 0.001, one-way ANOVA. Effects of BET inhibitors on parental and 5FU-TP JSC15-3 (**C**) and JSC18-1 (**D**) cell growth. The 5FU-TP cells were prepared by treating the respective parental cells with 3 µmol/L (JSC15-3) or 1 µmol/L (JSC18-1) 5-FU for 6 days. Then, the cells were reseeded and treated with the indicated concentrations of OTX015 (left) and I-BET-762 (right) for 6 days. Experiments were conducted with six technical replicates and were repeated at least three times. *, *P* < 0.05; **, *P* < 0.01, one-tailed Mann–Whitney rank-sum test. **E,** Effects of BET inhibitors on expression of exogenous ALDH1A3 in JSC15-3 cells. Mock and ALDH1A3-O/E JSC15-3 cells were treated as described in A and subjected to qRT-PCR analysis. Experiments were conducted with three technical replicates and were repeated at least three times. **F,** Effects of BET inhibitors on mock and O/E-derived DTP cell growth. Cells were treated as described in C, and cell numbers were quantitated. Experiments were conducted with six technical replicates and were repeated three times. *, *P* < 0.05; **, *P* < 0.01; ***, *P* < 0.001; ****, *P* < 0.0001, one-way ANOVA. ns, not significant.

To elucidate the mechanism of 5-FU–induced generation of gastric cancer persister cells, we conducted chemical screening for compounds that interfered with the generation of ALDH1A3-overexpressing DTP cells. Using a chemical library of 95 compounds, including various inhibitors, we screened for compounds that additively suppressed gastric cancer cell proliferation in combination with 5-FU. For the hit compounds in the primary screening (70 compounds), we performed a secondary screening for compounds that suppressed 5-FU–induced GFP expression in ALDH1A3-GFP cells (Supplementary Table S3). As a result, we identified inhibitors of BET family proteins, OTX015/birabresib and I-BET-762/molibresib, as the most potent suppressors of 5-FU–induced GFP expression (Supplementary Fig. S4A). In fact, co-treated BET inhibitors significantly suppressed 5-FU–induced GFP (i.e., ALDH1A3) expression and reduced the residual cell number after the drug treatment. These data demonstrate that 5-FU and BET inhibitors exhibit additive effects on cell growth (Supplementary Fig. S4B and S4C). BET family proteins recognize histone acetylations, such as H3K27ac, and activate transcription. As shown in Fig. 4A, OTX015 and I-BET-762 inhibited 5-FU–induced ALDH1A3 expression in JSC15-3 and JSC18-1 cells. Furthermore, both inhibitors decreased the number of GFP-positive cells induced by 5-FU (Fig. 4B; Supplementary Fig. S4D and S4E) and CDDP (Supplementary Fig. S4F and S4G). These results indicated that ALDH1A3 expression in DTP cells is highly dependent on BET proteins. We also determined whether the drug combination shows additive or synergistic interaction. Importantly, 5FU-TP cells were more sensitive to BET inhibitors than the parental cells (Fig. 4C and D), whereas co-treatment with 5-FU and BET inhibitors had an additive effect, which was judged by the Loewe synergy score greater than 0 and smaller than 10 (Supplementary Fig. S4H–S4K; ref. 24). CDDP-TP cells were also more sensitive to BET inhibitors than parental cells (Supplementary Fig. S4L and S4M). We confirmed that OTX015 and I-BET-762 suppressed the expression of the BET inhibitor–sensitive gene *c-MYC* in parental and DTP cells (Supplementary Fig. S4N). To assess how BET protein–dependent ALDH1A3 expression contributes to DTP cell survival, we exogenously overexpressed (O/E) the *ALDH1A3* gene driven by the cytomegalovirus promoter. As shown in Fig. 4E, exogenous ALDH1A3 was stably overexpressed in these cells. Even in the presence of BET inhibitors, 5-FU–induced ALDH1A3 expression was maintained at levels comparable to those in 5-FU–treated control cells (Fig 4E, *bar* #8, #10, 12). Among mock cells, 5FU-TP cells were more sensitive to OTX015 than the parental cells (Fig. 4F). Conversely, among exogenous ALDH1A3 O/E cells, 5FU-TP cells did not show collateral sensitivity to OTX015. Collectively, these observations indicate that the BET protein–dependent induction of ALDH1A3 may be critically involved in DTP cell survival after 5-FU treatment.

### BRD4 is an Essential Regulator of ALDH1A3 Expression in Gastric Cancer DTP Cells

**FIGURE 5:**
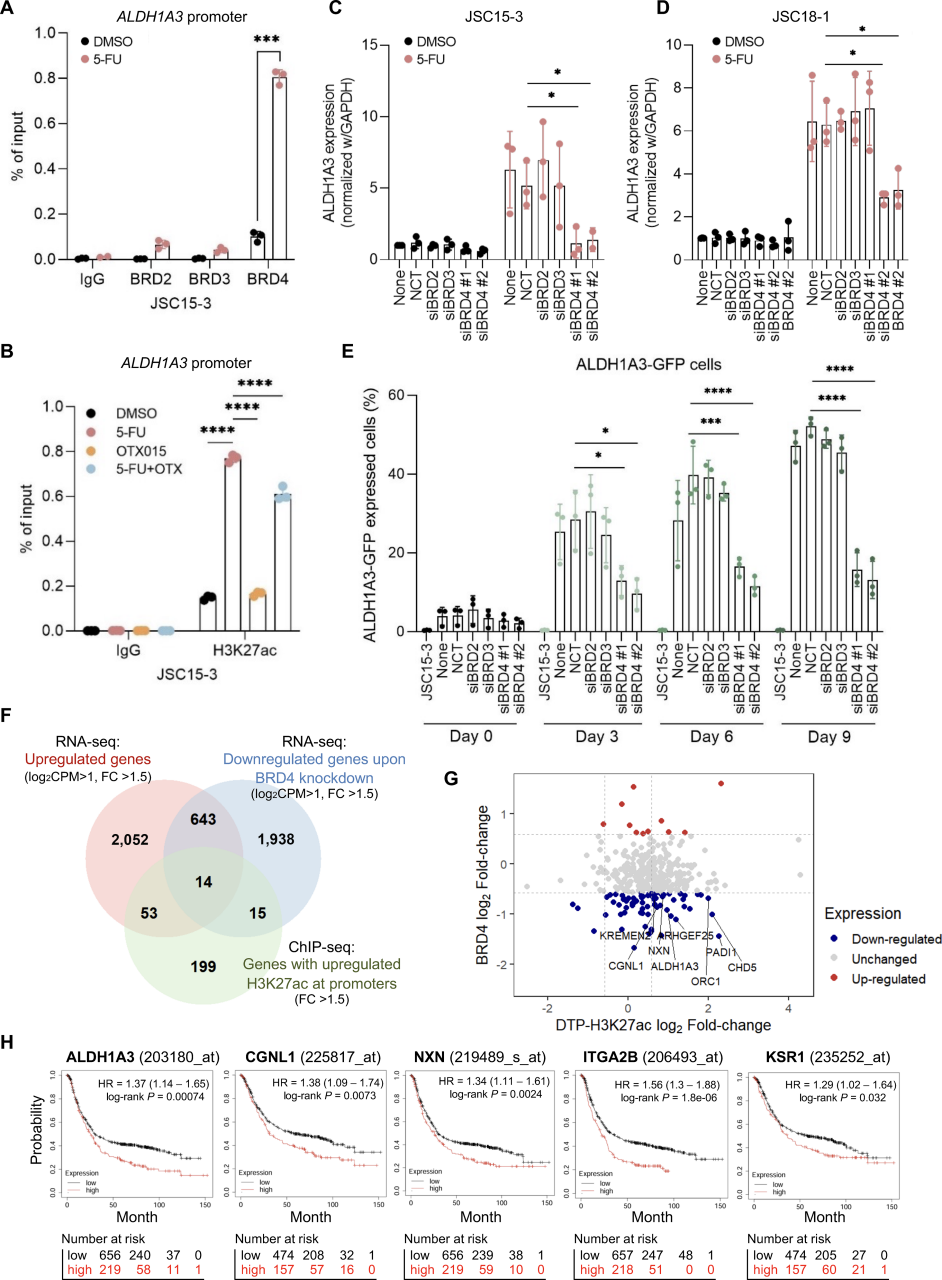
BRD4 mediates ALDH1A3 expression in DTP cells. **A,** ChIP-PCR analysis of BET proteins with the *ALDH1A3* promoter region. Cells were treated with 3 µmol/L 5-FU or DMSO for 5 days. Each bar represents the mean ± SD of three technical replicates. Experiments were repeated at least three times. ***, *P* < 0.001, two-sided *t* test. **B,** ChIP-PCR analysis of H3K27ac in the *ALDH1A3* promoter region in DTP cells. JSC15-3 cells were treated with 3 µmol/L 5-FU or DMSO and/or 1 µmol/L OTX015 for 5 days. Each bar represents the mean ± SD of three technical replicates. Representative data are shown. ****, *P* < 0.0001, one-way ANOVA. **C** and **D,** Effects of knockdown of BET family proteins on ALDH1A3 expression. Cells were transfected with either BRD2–4 or negative control (NCT) siRNAs for 24 hours. Then, the cells were treated with 3 µmol/L (JSC15-3; C) or 1 µmol/L (JSC18-1; D) 5-FU for 3 days and subjected to qRT-PCR analysis. Experiments were conducted with three technical replicates and were repeated at least three times. *, *P* < 0.05, one-way ANOVA. **E,** FACS analysis of BRD2–4-depleted ALDH1A3-GFP JSC15-3 cells and the parental JSC15-3 cells as a negative control for GFP detection. Cells were transfected with siRNAs, treated with 5-FU for the indicated days, and then subjected to flow cytometry. Experiments were repeated at least three times. *, *P* < 0.05; ***, *P* < 0.001; ****, *P* < 0.0001, one-way ANOVA. None, no siRNA transfected. **F,** Venn diagram of 2,762 upregulated genes (>1.5-fold change) and 281 genes with upregulated H3K27ac signals (>1.5-fold change) in DTP cells and 2,610 downregulated genes (>1.5-fold change) in BRD4-depleted cells treated with 5-FU. **G,** Scatter plot of the fold change in H3K27ac signals and expression upon BRD4 depletion. **H,** Kaplan–Meier plots for overall survival of patients with gastric cancer treated by surgery with or without adjuvant chemotherapy, who were stratified into high and low expression groups in accordance with the top three triple overlapped genes in F.

BET family proteins are epigenetic readers of histone lysine acetylation and are crucial oncogenic transcriptional co-activators in several types of cancers (25). In particular, BRD2–4 are essential members of the BET protein family, which have emerged as rational anticancer targets, together with pan-BET inhibitors such as OTX015 and I-BET-762 (26). To elucidate the BET protein involved in ALDH1A3 expression, we performed a ChIP-PCR assay using JSC15-3 cells. We observed selective BRD4 recruitment to the *ALDH1A3* promoter in DTP cells (Fig. 5A). Consistent with the role of BET proteins as readers of histone lysine acetylation marks, OTX015 only marginally decreased 5-FU–induced H3K27 acetylation (Fig. 5B), suggesting that BET inhibitors block the recruitment of BRD4, but not the H3K27ac mark on the *ALDH1A3* promoter. To clarify the differential requirements of BET proteins for ALDH1A3 induction in DTP cells, we knocked down each BET protein using siRNAs. The knockdown efficiency of each siRNA was verified at mRNA (Supplementary Fig. S5A) and protein (Supplementary Fig. S5B) levels. As for the discrepancy between BRD2 mRNA and protein levels in JSC18-1 cells treated with siBRD2, Western blot analysis of BRD2 gave double bands. Among them, the smaller one was resistant to the siRNA-mediated knockdown. Thus, siRNA-resistant signal in qRT-PCR might be derived from this minor isoform. BRD4 knockdown preferentially diminished 5-FU–induced ALDH1A3 expression (Fig 5C and D). Time-course monitoring of ALDH1A3-GFP cells confirmed that BRD4 knockdown significantly suppressed the 5-FU–induced accumulation of ALDH1A3^high^ cells (Fig. 5E; Supplementary Fig. S5C). These observations indicate that BRD4 recruitment to the *ALDH1A3* promoter with enriched H3K27ac is essential for ALDH1A3 induction in gastric cancer DTP cells.

As described above, H3K27ac elevation is a genome-wide phenotype of DTP. To assess the global role of BRD4 and H3K27ac-dependent gene expression in DTP cells, JSC15-3 cells were treated with BRD4 siRNA in the presence or absence of 5-FU, and RNA-seq analyses were performed. Among the DTP cell–specific genes with elevated H3K27ac marks (67 genes, Fig. 3F), expression of 14 genes was dependent on BRD4 (Fig. 5F). Notably, among the 14 genes, ALDH1A3 was included in the top genes that showed a high dependence on BRD4 (Fig. 5G). Kaplan–Meier plots of 631 patients with gastric cancer after chemotherapy [GEO repository (https://www.ncbi.nlm.nih.gov/geo/): GSE14210, GSE15459, GSE22377, GSE29272, GSE51105, and GSE62254] showed that the patient group with high expression of these DTP genes, such as ALDH1A3, CGNL1, NXN, ITGA2B, and KSR1, exhibited worse overall survival than the low expression group (Fig. 5H; ref. 27). These data suggest the potential significance of BRD4-mediated DTP gene regulation in the clinical outcome of gastric cancer.

### Therapeutic Effect of a BET Inhibitor in a Gastric Cancer PDC Xenograft Model

**FIGURE 6:**
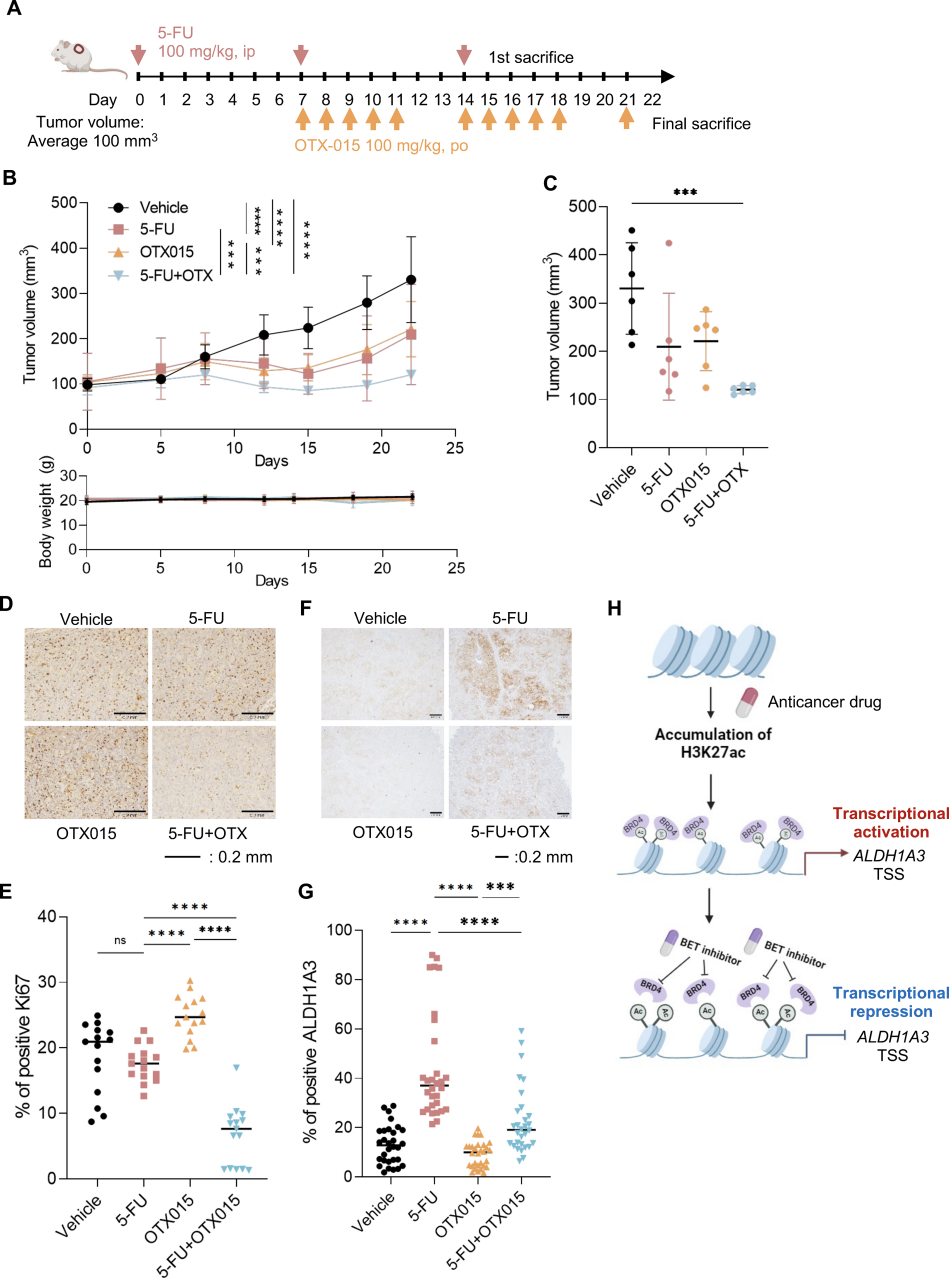
Combined effect of 5-FU and OTX015 on tumor growth *in vivo.***A,** Experimental design of combination therapy with 5-FU and OTX015 in xenografted mice. JSC15-3 cells were subcutaneously injected into nude mice. After 7 days, mice were injected intraperitoneally with 100 mg/kg 5-FU at days 0, 7, and 14 and treated orally with 100 mg/kg OTX015 at days 7–11, 14–18, and 21 or both drugs. **B,** Tumor volume (top) and mouse body weight (bottom). Error bars represent the SD. ***, *P* < 0.001; ****, *P* < 0.0001, one-way ANOVA. **C,** Average tumor volume on day 21. *N* = 6; ***, *P* < 0.001, one-way ANOVA. **D,** IHC of Ki-67. Images are representative of five biological replicates. **E,** Quantitative data of D. IHC of ALDH1A3 (**F**) and its quantitative data (**G**). ***, *P* < 0.001; ****, *P* < 0.0001, one-way ANOVA. **H,** Schematic view of anticancer drug-induced ALDH1A3 expression in DTP cells in H3K27ac- and BRD4-dependent manners. TSS: transcription start site.

Next, we investigated the therapeutic effect of a BET inhibitor with 5-FU in a JSC15-3 xenograft mouse model. NOD-SCID mice were subcutaneously injected with JSC15-3 cells and treated with 5-FU (100 mg/kg, i.p.), OTX015 (100 mg/kg, orally), or both (Fig. 6A). As shown in Fig. 6B top and C, the combination of 5-FU and OTX015 most significantly suppressed tumor growth compared with the vehicle and 5-FU or OTX015 alone. During the treatment, no or only marginal body weight loss was observed (Fig 6B, bottom). IHC of Ki-67 in endpoint samples revealed the suppression of cell proliferation by the combination of 5-FU and OTX015 (Fig. 6D and E). While 5-FU upregulated ALDH1A3 expression (Fig. 6F and G), its gene amplification at the genomic level was not observed in the 5-FU–treated tumors (Supplementary Fig. S6A). Importantly, 5-FU–induced upregulation of ALDH1A3 was repressed by OTX015 (Fig. 6F and G). We also examined mRNA levels of marker genes for cancer stemness, epithelial–mesenchymal transition, and drug resistance in xenograft tumor tissues and found no difference between the vehicle and 5-FU–treated groups (Supplementary Fig. S6B). Taken together, these observations indicate that BET inhibitors, which potentially interfere with BRD4 recruitment to the *ALDH1A3* promoter, decrease DTP cell survival and enhance chemotherapeutic efficacy against gastric tumors *in vivo* (Fig. 6H).

## Discussion

In this study, we demonstrated epigenetic induction of ALDH1A3-overexpressing gastric cancer DTP cells after chemotherapy. In terms of residual cancer cells after drug treatment, two potential mechanisms have been considered: selection of a preexisting small subpopulation of intrinsically drug-resistant cells, such as cancer stem cells, and induction of cancer cells with a drug-resistant phenotype after drug stimuli. Our current observations are consistent with recent studies of other cancers showing that the DTP state is an induced phenotype rather than a selection of preexisting drug-resistant cells (5, 6, 28). DTP cells are slow-proliferating diapause-like cells that often express cancer stem cell markers (29–35). However, a recent report showed that DTP cells include cycling and noncycling cells, which develop distinct transcriptional and metabolic programs (6). In the gastric cancer PDCs examined in this study, most cells converted to ALDH1A3^high^ cells after 5-FU treatment, whereas ALDH1A3 induction remained heterogeneous in the cancer cell population (Fig. 2G). Further studies are needed to clarify which cellular signals determine heterogenous DTP inducibility. As for the coexistence of small population of ALDH1A3^low^ cells in the ALDH1A3^high^ cells at day −1, this would be a technical limitation due the nature of cell-cell attachment between the small number of ALDH1A3^high^ cells and major fraction of ALDH1A3^low^ cells in initial cell population. Despite the coexistence of small number of ALDH1A3^low^ cells in the sorted ALDH1A3^high^ cells, these results still clearly showed the unstable nature of ALDH1A3^high^ cells in the absence of 5-FU (Fig 2G). We tried sorting of GFP-positive cells from DTP cells, but the number of collected cells was too small to perform subsequent drug sensitivity test. While we observed a tendency of 5-FU resistance in the 5FU-TP cells from ALDH1A3^low^ cells, there was no statistically significant difference (Supplementary Fig S2I, bottom). This could potentially be due to a relatively low average ALDH1A3 expression level in the 5FU-TP cells from ALDH1A3^low^ cells, when compared with the 5FU-TP cells from ALDH1A3^high^ cells (Fig. 2G).

Epigenetic regulation, such as histone modification and regulation by related factors, plays a pivotal role in cellular plasticity. In gastric cancer DTP cells, we found that an increase in H3K27ac and BRD4, acetylated histone-dependent modifiers, was critical for the expression of ALDH1A3, a DTP survival factor. Concerning the epigenetic regulation of ALDH1A3 expression, feed-forward regulation between a histone demethylase, KDM4C, and ALDH1A3 has been reported in gastric cancer stem cells (36). In addition, ALDH1A3 promotes H3K27ac in pulmonary arterial hypertension by converting acetaldehyde to acetate (36–38). These reports suggest additional cross-talk between ALDH1A3 and epigenetic reprogramming in DTP cells. Moreover, a global decrease in H3K27me3 or accumulation of H3K9me3 has been observed in triple-negative breast cancer cells after chemotherapy and in lung cancer cells after treatment with a tyrosine kinase inhibitor of the EGFR, respectively. These observations represent cell context–dependent regulation of DTP cell plasticity.

The initial signaling pathway for epigenetic reprogramming of gastric DTP cells remains elusive, particularly the global elevation of H3K27ac- and BRD4-dependent ALDH1A3 induction. We evaluated the ChIP-seq data and particularly we focused the genes with highly elevated H3K27ac signals in DTP cells (fold change >1.5 in 5-FU–treated cells/DMSO control). Still, however, as shown in Fig. 3F, the overlapped gene number was small. According to several previous reports, such small overlaps between transcriptionally upregulated genes and the genes with a certain open chromatin mark were also observed (39, 40). This could potentially be due to the existence of multiple gene expression regulation apart from epigenetic histone modifications, as well as to the potential cooperation of multiple histone modifications in epigenetic regulation of gene expression. In other cancers, several mechanisms have been proposed to induce the DTP phenotype or related histone modifications, such as integrated stress response (28), reactive oxygen species (13, 32, 41, 42), histone acetyltransferase (HAT) activity (43, 44), and DNA damage (43, 45). In our ALDH1A3-GFP knock-in model, GFP induction was suppressed by BET inhibitors but not by HAT inhibitors or other inhibitors of epigenetic regulators (Supplementary Fig. S4A; Supplementary Table S3), suggesting the involvement of other pathways. Kaplan–Meier analysis revealed that the triple overlapping genes among upregulated genes in DTP cells, downregulated genes in BRD4-depleted cells, and genes with 5-FU–induced H3K27ac peaks (14 genes in Fig. 5F–H) were closely related to the clinical outcome. Further analyses are required to identify the detailed mechanism of the initial histone modification after anticancer drug treatment.

Accumulating evidence indicates that BET inhibitors are rational agents that target cancer. BRD4 binds to H3K27ac in gene enhancers and promoter loci as an epigenetic reader and recruits mediator complexes (46). In cancer cells, BRD4 regulates the expression of oncogenic gene *c-MYC* (47–49) and DNA damage response factors (50). In gastric cancer PDCs, we confirmed that BET inhibitors suppressed *c-MYC* expression in parental and DTP cells (Supplementary Fig. S4N), whereas *ALDH1A3* expression and cell survival were more preferentially suppressed in DTP cells than in parental cells. These results provide additional support for the therapeutic application of BET inhibitors in drug-resistant gastric cancer cells.

In summary, this study highlights that the perturbation of DTP-related epigenetic changes is a novel therapeutic strategy to eliminate gastric cancer DTP cells.

## Supplementary Material

Supplementary Materials and Methods Supplementary Materials and Methods

Supplementary Tables S1 Primer sequences used in the study

Supplementary Table S2 Chemical compounds used for the screening

Supplementary Table S3 Results of secondary screening for compounds that suppress 5-FU-induced GFP expression in ALDH1A3-GFP cells

Supplementary Figure S1 Decreased sensitivity of cisplatin-tolerant persister cells to cisplatin and reversibility of ALDH1A3 expression in 5-fluorouracil-tolerant persister cells

Supplementary Figure S2 Establishment of ALDH1A3-GFP knock-in JSC15-3 cells to evaluate ALDH1A3 induction after anticancer drug treatment

Supplementary Figure S3 Distribution and implication of H3K27ac marks in 5-FU-tolerant persister JSC15-3 cells

Supplementary Figure S4 BET inhibitors suppress ALDH1A3 upregulation and preferentially inhibit DTP cell growth

Supplementary Figure S5 BRD4 knockdown suppresses 5-FU-induced ALDH1A3 upregulation.

Supplementary Figure S6 Estimation of ALDH1A3 gene amplification and the levels of biomarkers in 5-FU treated xenograft tumors
