# Supplementary material for: Pharmacologic Targeting of Histone H3K27 Acetylation/BRD4-dependent Induction of ALDH1A3 for Early-phase Drug Tolerance of Gastric Cancer
Source: Cancer Res Commun. 2024 May 20;4(5):1307–20. doi: 10.1158/2767-9764.CRC-23-0639 (PMC11104289; doi:10.1158/2767-9764.CRC-23-0639)
Supplement: Supplementary Materials and Methods [file crc-23-0639-s01.docx]

**Pharmacological targeting of histone H3K27 acetylation/BRD4-dependent induction of ALDH1A3 for early-phase drug tolerance of gastric cancer**

Jin Lee *et al.*

**Supplementary Materials and Methods**

**Immunohistochemistry**

Paraffin-embedded sections were deparaffinized by xylene. To replace ethanol for xylene, sections were immersed in 70% ethanol and distilled water (DW). Using 1:10 diluted DAKO REAL Target Retrieval Solution (DAKO), antigen activation was conducted for 30 min. After washing with DW, sections were immersed in 0.3% hydrogen peroxide in methanol for 10 min at room temperature. Washed sections with Tris-buffered saline (TBS) and 0.1% Tween-20 (Nacalai Tesque) were blocked for 10 min at room temperature with Blocking One Histo (Nacalai Tesque). Each section was detected with primary antibodies in TBST overnight at 4℃. After washing with TBST, sections were stained with EnVision™+ Dual Link System-HRP (Dako) for Ki67, N-Histofine® Simple Stain MAX PO (Multi) (424151, Nichirei, Tokyo, Japan) for ALDH1A3 for 30 min at room temperature. For color reaction, sections were treated with the Liquid DAB+ substrate-chromogen system (Dako) for 1 min at room temperature. After washing with water, sections were stained with hematoxylin for 10 sec and washed with DW. These sections were dehydrated with 70% ethanol, 100% ethanol, and xylene. Finally, each section was sealed with Mount-quick (Daido Sangyo). The following antibodies were used for immunocytochemistry: Ki67 (1:500, Abcam, ab15580), ALDH1A3 (1:500, GeneTex, GTX633822). Analysis was performed with Qpath (version 0.5.0).

**Western blot analysis**

After collecting cell lysates, proteins were quantified using the Bradford method. Lysates were electrophoresed on a 10% SDS-polyacrylamide gel for 1.5 hours and then electrophoretically transferred onto an Immobilon membrane (Nacalai Tesque) for 1.5 hours. The membrane was incubated with Tris-buffered saline (TBS) containing 5% dry skim milk and 0.1% Tween-20 (Nacalai Tesque). The membrane was incubated with the primary antibody (described below) diluted in 5% dry skim milk in TBS containing 0.1% Tween-20 (TBST) overnight at 4°C, washed three times in TBST, and incubated with the secondary antibody in 5% dry skim milk in TBST for 1 hour at room temperature (20-25°C). Then, the membrane was washed three times in TBST. Protein bands were detected with ECL™ Western Blotting Detection Reagents (Cytiva). Data were visualized using an Amersham ImageQuant 800 (Cytiva).

**Mouse xenograft model**

Animal procedures were performed in accordance with protocols approved by the Japanese Foundation for Cancer Research Animal Care and Use Committee. To evaluate the therapeutic effect of 5-fluorouracil (5-FU) and OTX015, JSC15-3 cells (2 × 10^6^/site) were suspended in 700 μL Hank’s balanced salt solution with 700 μL Matrigel (Corning) and subcutaneously implanted into 5-week-old female nude mice (Charles River Laboratories Japan) (n=6 per group). When the average tumor volume reached 100 mm^3^, mice were separated into four groups (vehicle, 5-FU, OTX015, and 5-FU and OTX015 combination). Next, 5-FU (250 mg) was diluted with PBS to 50 mg/mL, and OTX015 was solubilized with 5% dimethyl sulfoxide (DMSO), 40% PEG300, 5% Tween 80, and 50% saline. Mice were treated as follows: (1) vehicle (n=6); (2) 100 mg/kg 5-FU was intraperitoneally injected once a week from day 0 to 14 (n=6); (3) 100 mg/kg OTX015 was administrated orally five times a week from day 7 to 22 (n=6); (4) 100 mg/kg 5-FU was injected once a week from day 0 to 14 and 100 mg/kg OTX015 was sequentially administered five times a week from day 7 to 22 (n=6). The tumor volume was calculated as described above. Measurements were performed using a digital caliper every 3 or 4 days. We also measured the mouse body weight to estimate adverse effects of the treatment.

**Chemical compounds**

Cisplatin (CDDP) was purchased from Enzo Biochem and 5-FU was purchased from Sigma-Aldrich. Trichostatin A was purchased from Wako Pure Chemical Industries. EPZ5676, OTX015/birabresib, and I-BET-762/molibresib were purchased from Selleckchem. EPZ6438/tazemetostat and SP2509 were purchased from MedChemExpress.

**qPCR**

JSC15-3 and JSC18-1 cells were treated with DMSO or 3 μM 5-FU for 5 days. Genomic DNA (gDNA) of cultured cells and *in vivo* samples were collected using DNeasy Blood & Tissue kit (Qiagen, Venlo, Netherlands). Collected gDNA was quantitated by NanoDrop (Thermo Fisher Scientific, Waltham, MA). qPCR was performed using the same concentration of gDNA samples with Power SYBR Green PCR Master Mix (Applied Biosystems, Waltham, MA) in a StepOnePlus real-time PCR system (Thermo Fisher Scientific). Primers used are shown in Supplementary Table 1. GAPDH (Exon) was used as the internal control to normalize the data.

**CellTiter-Glo assay**

JSC15-3 and JSC18-1 cells were treated with DMSO or 3 μM 5-FU. After 5 days, cells were reseeded at 2,000 cells per well, respectively, in a 96-well plate. Cell viability was analyzed using CellTiter-Glo^®^ 2.0 Cell Viability Assay (Promega). For the quantitation, the luminescent signal was measured by time-course (0, 3, 6, and 9 days). Six replicates were set at each time point. Each experiment was performed at least three times to confirm the reproducibility.

**Cell proliferation assay (MTT assay)**

To evaluate cell proliferation, JSC15-3 and JSC18-1 cells were seeded at 1,000 and 2,000 cells per well, respectively, in a 96-well plate. Then, the cells were treated with various concentrations of the drugs. Six replicates were included for each time point. MTT [3-(4,5-dimethyl-2-thiazolyl)-2,5-diphenyltetrazolium bromide] (Nacalai Tesque) was added to cells at a final concentration of 0.8 mg/mL. Cells were incubated for 4 hours, and then formazan crystals were dissolved in DMSO. Optical densities at 570 and 630 nm were measured using an xMark microplate spectrophotometer (Bio-Rad). Each experiment was performed at least three times to confirm reproducibility. To compare drug sensitivities of parental and drug-tolerant persister (DTP) cells, the cells were treated with each anticancer drug at the IC_50_ value for the parental cells (i.e., 3 μM 5-FU for JSC15-3 cells and 1 μM 5-FU for JSC18-1 cells; 10 μM CDDP for JSC15-3 cells and 1 μM CDDP for JSC18-1 cells) for 5 days. The surviving cells were collected as DTP cells, which were subjected to further MTT assays and used for other experiments.

**Trans-well invasion assay**

For trans-well migration studies, the CytoSelect^TM^ Cell Migration and Invasion Assay (Cell Biolabs, San Diego, CA) was used, and samples were processed according to the manufacturer’s instructions. First, cells were untreated or treated with 3 μM 5-FU. Then, 5 × 10^5^ cells were suspended in serum-free medium and plated on the upper side of the membrane with ECM matrix (basement membrane, 8-μm pore size) whereas the medium containing 10% fetal bovine serum was added to the lower chamber. MDA-MB-231 cells (RRID:CVCL_0062) were used as a positive control. After incubation for 48 h, the medium was aspirated, and non-invasive cells were removed with a cotton-tipped swab. Each sample was stained with cell stain solution, rinsed in DW, and dried. Then, the extraction solution was added and transferred from each sample to a 96-well microtiter plate. Finally, OD 560 nm was measured in a plate reader.

**Chromatin immunoprecipitation (ChIP) assay**

Chromatins were lysed with the lysis buffers, and DNA was digested to approximately 150–900 bp with 0.5 µL micrococcal nuclease and sonication. After centrifugation, the supernatant was collected, and the DNA concentration was quantitated by Qubit Fluorometric Quantification using a Qubit™ dsDNA Quantification Assay Kit (Thermo Fisher Scientific). Agarose gel electrophoresis was performed to verify the DNA fragment size. Digested samples (5–10 µg) were incubated with an anti-histone H3 antibody as a positive control, IgG as a negative control, or anti-H3K4me3, -H3K27ac, -H3K27me3, -H3K9me3, -BRD2, -BRD3, and -BRD4 antibodies overnight at 4°C. The immunocomplexes were mixed with Magnetic Beads (Cell Signaling Technology, 9003). After washing the immunoprecipitants with low and high salt buffers, bead-bound DNA was eluted using DNA elution buffer and subjected to ribonuclease A and protein K treatments. Crosslinks were reversed by incubation at 65°C overnight, and ChIP DNA was purified in DNA Purification Columns and Collection Tubes. qPCR was performed as described in the RT-qPCR section. Results were normalized to the input DNA and then subjected to Illumina ChIP-Seq library construction.

**ChIP-seq data analysis**

ChIP samples were subjected to ChIP-seq at Gene Bay. The sequence library was sequenced with a NovaSeq6000 (Illumina). Quality of raw FASTQ files was checked by FastQC (version 0.11.9) and adapters were removed using Trimmomatic (version 0.39) (RRID:SCR_011848). Reads were aligned to GRCh38 using bwa mem (version 0.7.17). Alignments were sorted (samtools version 1.15.1) and NarrowPeaks (RRID:SCR_012924) were called using MACS3 (version 3.0.0b3). Bed files were converted from NarrowPeaks using bedtools (version 2.27.1, RRID:SCR_006646). Each peak was annotated using ChIPseeker (version 1.34.1) and ChIPpeakAnno (version 3.32.0). We converted bw files to Bigwig files of normalized counts per million using deeptools bamCoverage (version 3.5.1) and visualized the peaks with Integrative Genome Viewer (IGV) (version 2.16.0). To distribute read signals, plotHeatmap from upstream 1.5 kb to downstream 1.5 kb was generated by DiffBind (version 3.8.4). To evaluate RNA-seq and ChIP-seq data by expression changes, differential binding analysis of H3K27ac signals was performed using csaw (version 1.32.0) in R package.

**Small interfering RNA (siRNA) experiments**

Silencer Select siRNAs against BRD2 (4427037, s12070), BRD3 (4427038, s15544), BRD4 #1 (4427038, s23903), and BRD4 #2 (4427038, s23901), and Silencer™ Select Negative Control No. 1 siRNA (4390843) were purchased from Thermo Fisher Scientific. Cells were seeded at 1 × 10^5^ cells/mL in a six-well plate and transfected with 20 μM siRNA using RNAiMAX Transfection Reagent (Thermo Fisher Scientific) and Opti-MEM in accordance with the manufacturer’s instructions. A cell lysate was prepared after 48 hours, and knockdown efficacy was examined by RT-qPCR and western blot analysis as described above.

**Antibodies**

The following antibodies were used for western blot analysis: anti-ALDH1A3 (1:2,000, GeneTex, GTX633822), -GAPDH (0.1 µg/mL, FL335, Santa Cruz Biotechnology), -BRD2 (1:1,000, Proteintech, 22236-1-AP), -BRD3 (1:1,000 Active motif, 61489), and -BRD4 (1:1,000, Cell Signaling Technology, 13440). The following antibodies were used for immunocytochemistry: anti-Ki-67 (1:500, Abcam, ab15580) and -ALDH1A3 (1:500, GeneTex, GTX633822). The following antibodies were used for ChIP assays: anti-histone H3 (1:50, Cell Signaling Technology, 4620S), normal rabbit IgG (0.1 μg/mL, Cell Signaling Technology, 2729S), anti-H3K27ac (1:100, Abcam, ab4729), -H3K4me3 (1:80, Milli pore, 07-473), -H3K27me3 (1:50, Cell Signaling Technology, 9733), and -H3K9me3 (1:80, Millipore, 17-10242). Antibodies against BRD2–4 (1:50) were the same as those for western blot analysis.

**Kaplan-Meier Plotter**

The Kaplan-Meier Plotter (http://kmplot.com/analysis/index.php?p=service&cancer=gastric) was used to measure the prognostic value of the overlapped genes in Fig. 5F (upregulated genes, genes with upregulated H3K27ac at promoters, and downregulated genes upon BRD4 knockdown). The Kaplan-Meier plots were made using the gene expression data and survival information of 875 gastric cancer patients: Gene Expression Omnibus (GEO) GSE14210, GSE15459, GSE22377, GSE29272, GSE51105, and GSE62254. To evaluate the overall survival (OS), patients were divided into two groups according to the basis of the upper and lower quartile gene expression (i.e., upper 75% and lower 25%, respectively). The Kaplan-Meier survival curves were analyzed using a log-rank test. Hazard ratios (HR) along with 95% confidence intervals (CI) were calculated, with statistical significance set at *P* < 0.05.
