# Supplementary material for: Pharmacologic Targeting of Histone H3K27 Acetylation/BRD4-dependent Induction of ALDH1A3 for Early-phase Drug Tolerance of Gastric Cancer
Source: Cancer Res Commun. 2024 May 20;4(5):1307–20. doi: 10.1158/2767-9764.CRC-23-0639 (PMC11104289; doi:10.1158/2767-9764.CRC-23-0639)
Supplement: Supplementary Figure S1 — Decreased sensitivity of cisplatin-tolerant persister cells to cisplatin and reversibility of ALDH1A3 expression in 5-fluorouracil-tolerant persister cells [file crc-23-0639-s05.pdf]

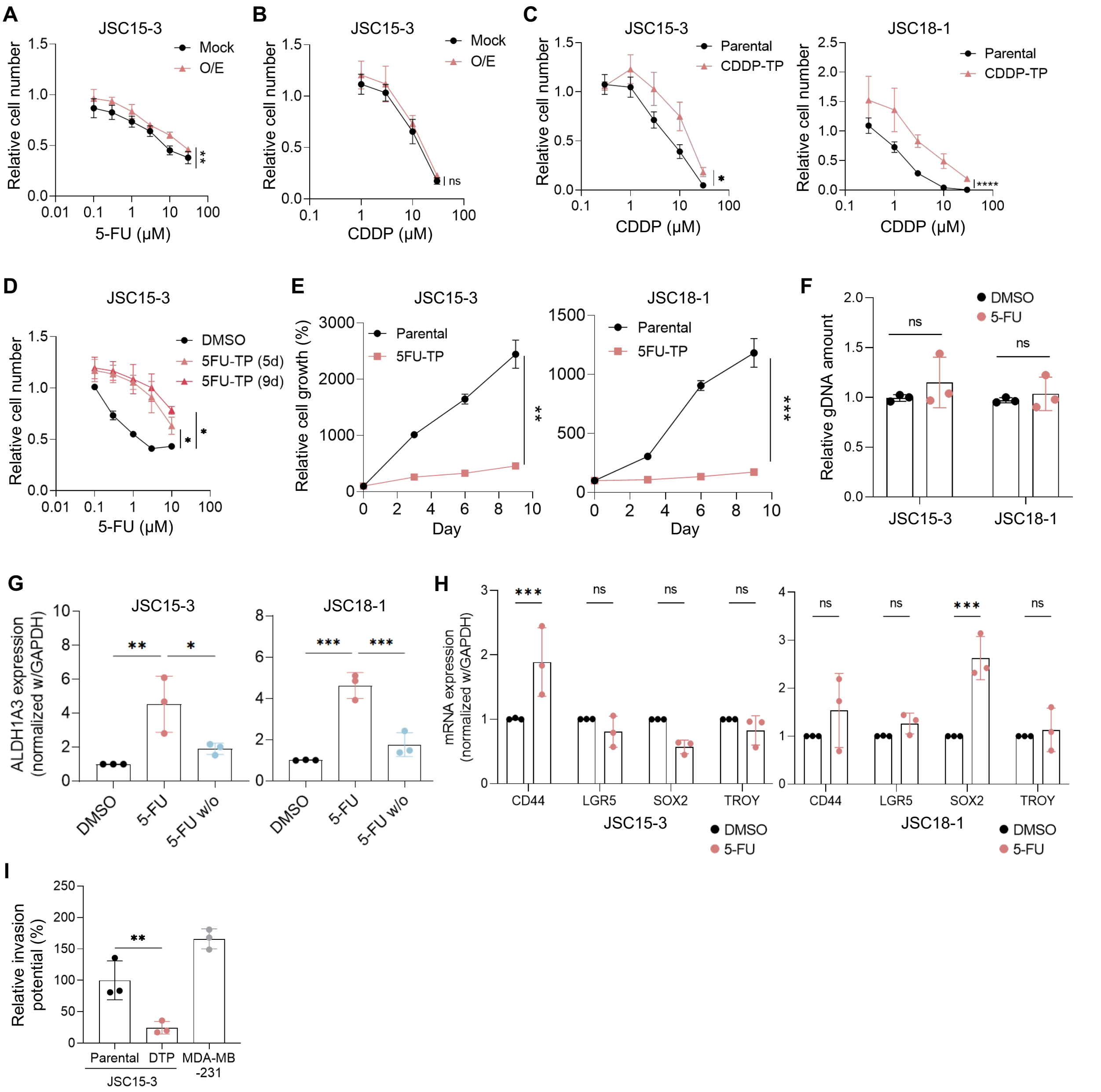

Supplementary Fig. S1 Decreased sensitivity of cisplatin-tolerant persister cells to cisplatin and reversibility of *ALDH1A3* expression in 5-fluorouracil-tolerant persister cells

A. Sensitivity of ALDH1A3-overexpressing (O/E) cells to 5-FU. Control (Mock) and ALDH1A3 O/E cells were treated with 5-FU at the indicated concentrations for 6 days. **B.** Sensitivity of ALDH1A3 O/E cells to cisplatin (CDDP). Mock and ALDH1A3 O/E cells were treated with CDDP at the indicated concentrations for 6 days. **C.** Effect of CDDP on CDDP-tolerant persister (CDDP-TP) cells. Parental JSC15-3 (left) and JSC18-1 (right) cells and their CDDP-TP cells were treated with CDDP at the indicated concentrations for 6 days. Each point represents the mean ± standard deviation of six technical replicates. Data are representative of at least three independent experiments. *$p<0.05$, **$p<0.01$, ****$p<0.0001$, two-tailed *t*-test. **D.** Sensitivities of 5FU-TP cells at day 5 and day 9 to 5-FU. Cells were treated with 5-FU at the indicated concentrations for 5 days. **E.** Comparison of cell growth between the parental and 5FU-TP cells, which were prepared by treating the parental cells with 5FU for 5 days. **F.** Evaluation of *ALDH1A3* gene copy number by genomic qPCR. Cells were treated with 3 μM (JSC15-3) and 1 μM (JSC18-1) 5-FU for 5 days. Genomic DNA samples with same concentration were subjected to qPCR analysis. **G.** Reversibility of *ALDH1A3* upregulation in DTP cells. JSC15-3 (left) and JSC18-1 (right) cells were treated with 3 μM (JSC15-3) and 1 μM (JSC18-1) 5-FU for 5 days and then cultured for an additional 14 days without 5-FU. RNAs were prepared and subjected to RT-qPCR analysis. **H.** mRNA transcript levels of cancer stem markers in DTP cells. Cells were prepared as in (G). **I.** Invasion assay. JSC15-3 cells (Parental, DTP cells), and MDA-MB-231 cells (positive control) that invaded through the Transwell were quantitated. DTP cells were prepared by treating the parental cells with 3 μM 5-FU for 5 days. Cells were seeded to Transwell plates, incubated for 48 hours, and the invaded cells were quantitated. Each sample was normalized by the parental triplicated samples. Experiments were performed with three technical replicates and repeated at least three times. *$p<0.05$, **$p<0.01$, ***$p<0.001$, one-way ANOVA.
