# Supplementary material for: Pharmacologic Targeting of Histone H3K27 Acetylation/BRD4-dependent Induction of ALDH1A3 for Early-phase Drug Tolerance of Gastric Cancer
Source: Cancer Res Commun. 2024 May 20;4(5):1307–20. doi: 10.1158/2767-9764.CRC-23-0639 (PMC11104289; doi:10.1158/2767-9764.CRC-23-0639)
Supplement: Supplementary Figure S2 — Establishment of ALDH1A3-GFP knock-in JSC15-3 cells to evaluate ALDH1A3 induction after anticancer drug treatment [file crc-23-0639-s06.pdf]

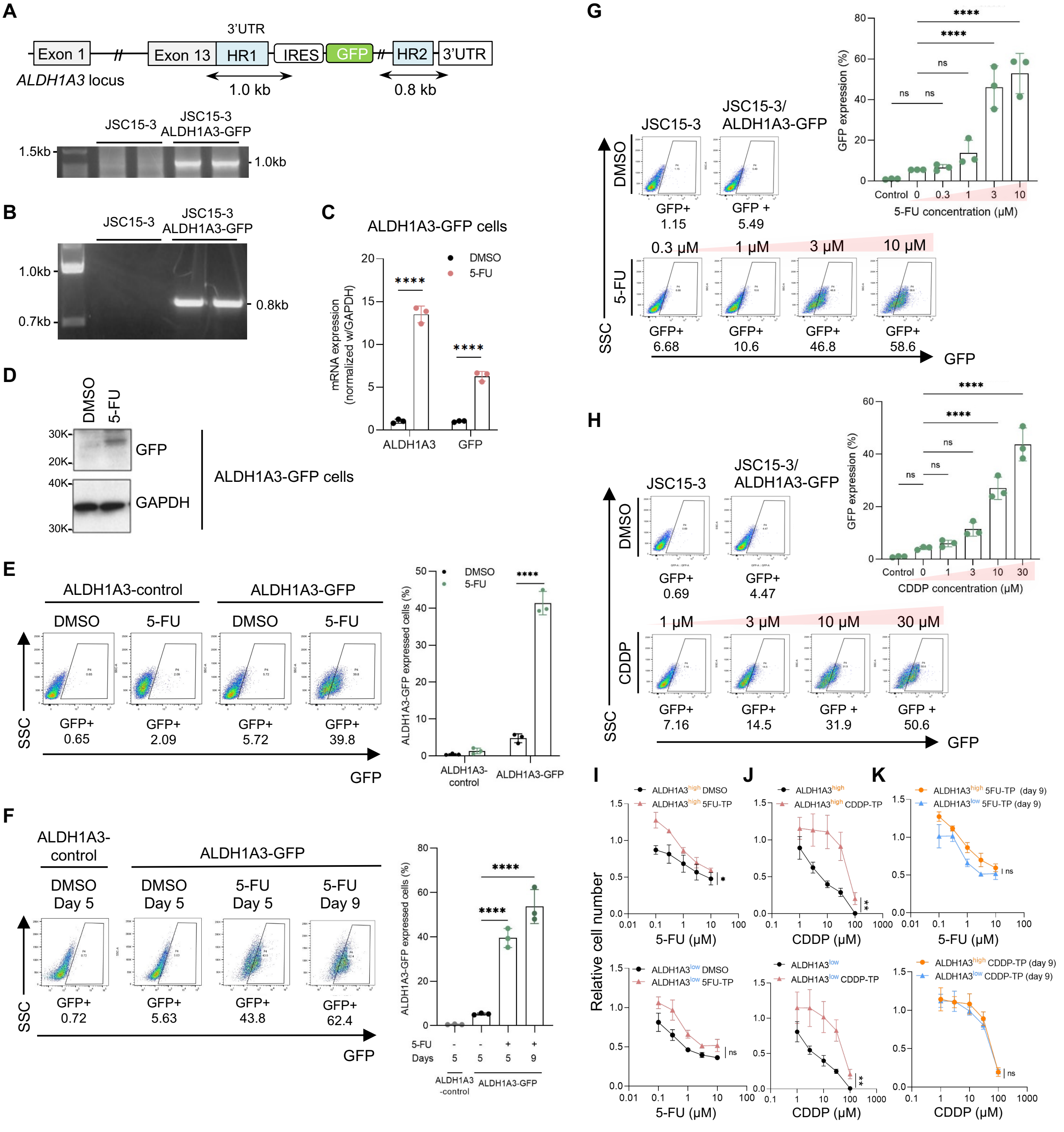

Supplementary Fig. S2 Establishment of ALDH1A3-GFP knock-in JSC15-3 cells to evaluate ALDH1A3 induction after anticancer drug treatment

A, B. Evaluation of gene knock-in of the ALDH1A3-GFP construct in JSC15-3 cells. Genomic DNA was prepared and subjected to PCR with primers at *ALDH1A3* exon 13 and IRES loci (A) and at loxP and *ALDH1A3* 3′-UTR (B), as depicted in Fig. 2C. Sequences of the amplified DNA fragments were confirmed by Sanger sequencing. **C.** ALDH1A3 and GFP induction in 5-FU-treated ALDH1A3-GFP JSC15-3 cells. Cells were treated with 3 μM 5-FU for 5 days and subjected to qRT-PCR. Experiments were performed with three technical replicates and were repeated at least three times. **D.** Cells treated as described in C were subjected to western blot analysis. **E.** FACS analysis of GFP expression in ALDH1A3-GFP cells. Cells were treated with 3 μM 5-FU or DMSO for 5 days. **F, G.** Time-course (F) and dose-dependent (G) evaluations of GFP expression in 5-FU-treated ALDH1A3-GFP cells. **H.** Dose-dependent evaluation of GFP expression in CDDP-treated ALDH1A3-GFP cells. In E–H, experiments were performed at least three times. ****p<0.0001, one-way ANOVA. **I.** The 5-FU sensitivity of 5FU-TP cells derived from sorted ALDH1A3^{high} (left) and ALDH1A3^{low} (right) cells as described in Fig. 2G. **J.** CDDP sensitivity of CDDP-TP cells derived from sorted ALDH1A3^{high} (left) and ALDH1A3^{low} (right) cells. **K.** Comparison of drug sensitivities of ALDH1A3^{high} DTP cells and ALDH1A3^{low} DTP cells (left: 5FU-TP, right: CDDP-TP) prepared for 9-day treatment with the drug. Experiments were performed with six technical replicates and repeated three times with equivalent results.
