# Supplementary material for: Pharmacologic Targeting of Histone H3K27 Acetylation/BRD4-dependent Induction of ALDH1A3 for Early-phase Drug Tolerance of Gastric Cancer
Source: Cancer Res Commun. 2024 May 20;4(5):1307–20. doi: 10.1158/2767-9764.CRC-23-0639 (PMC11104289; doi:10.1158/2767-9764.CRC-23-0639)
Supplement: Supplementary Figure S3 — Distribution and implication of H3K27ac marks in 5-FU-tolerant persister JSC15-3 cells [file crc-23-0639-s07.pdf]

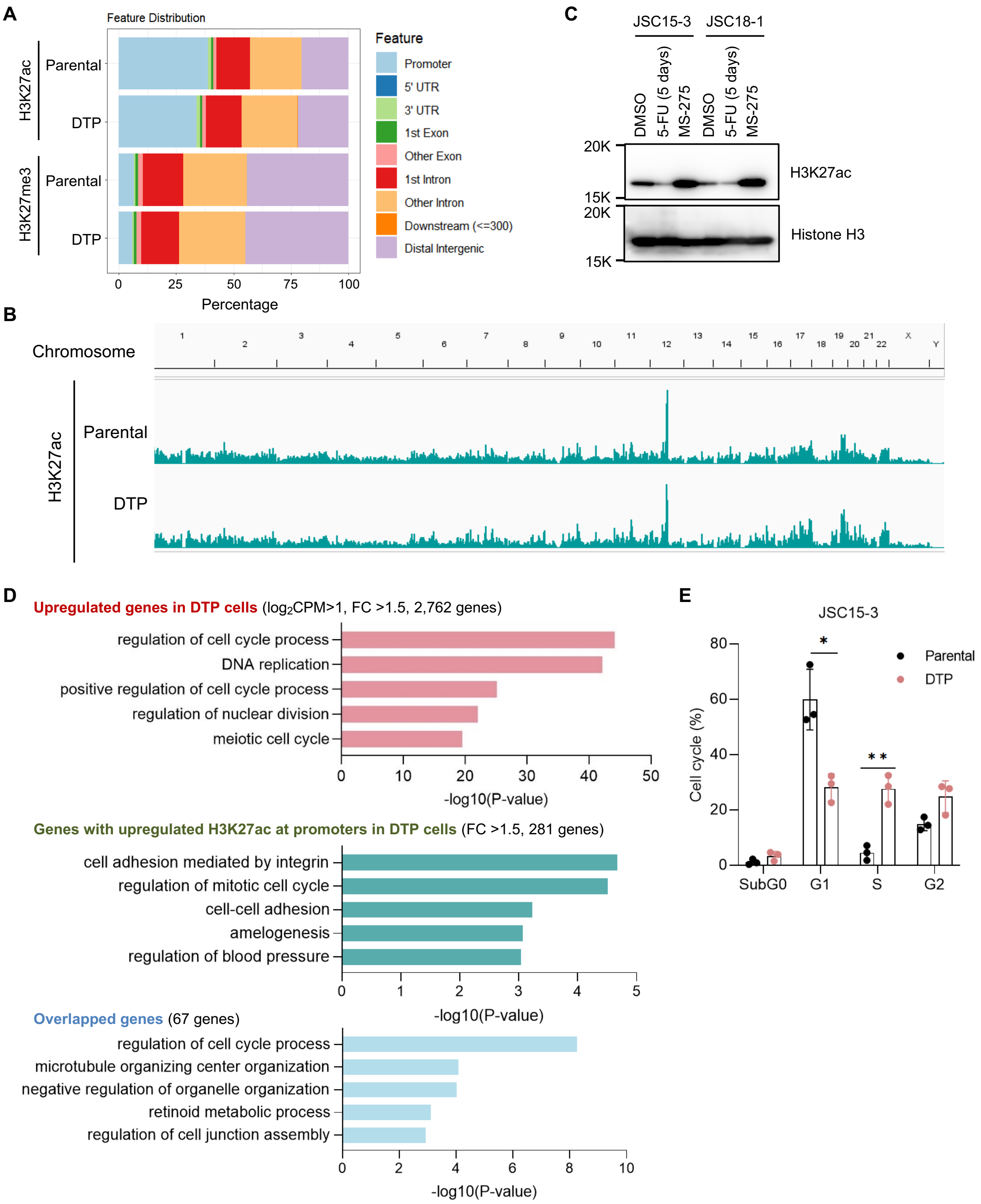

Supplementary Fig. S3 Distribution and implication of H3K27ac marks in 5-FU-tolerant persister JSC15-3 cells

A. H3K27ac and H3K27me3 peak distribution in gene features. ChIP seq data of parental and DTP JSC15-3 cells were analyzed using ChIPseeker, an R package (version 3.8.4). Each peak was annotated with "org.Hs.eg.db". **B.** Levels of H3K27ac in all chromosome regions of the parental and DTP cells demonstrated by IGV. **C.** Western blot analysis of total H3K27ac. Histone H3 was used as the control of histone extraction and loading. Cells were treated with 3 µM of 5-FU for 5 days or 3 µM of MS-275 as positive control for 24 hours. **D**. Clustered enrichment ontology categories derived from 2,762 upregulated genes in DTP cells (top), 281 genes with upregulated H3K37ac in DTP cells (middle), and 67 overlapped genes (Fig. 3F). **E.** Effect of 5-FU on the cell cycle distribution of JSC15-3 cells. Cells were treated with 3 µM 5-FU for 5 days and subjected to propidium iodide staining and flow cytometry. *p<0.05, **p<0.01, two-tailed t-test.
