# Supplementary material for: Pharmacologic Targeting of Histone H3K27 Acetylation/BRD4-dependent Induction of ALDH1A3 for Early-phase Drug Tolerance of Gastric Cancer
Source: Cancer Res Commun. 2024 May 20;4(5):1307–20. doi: 10.1158/2767-9764.CRC-23-0639 (PMC11104289; doi:10.1158/2767-9764.CRC-23-0639)
Supplement: Supplementary Figure S4 — BET inhibitors suppress ALDH1A3 upregulation and preferentially inhibit DTP cell growth [file crc-23-0639-s08.pdf]

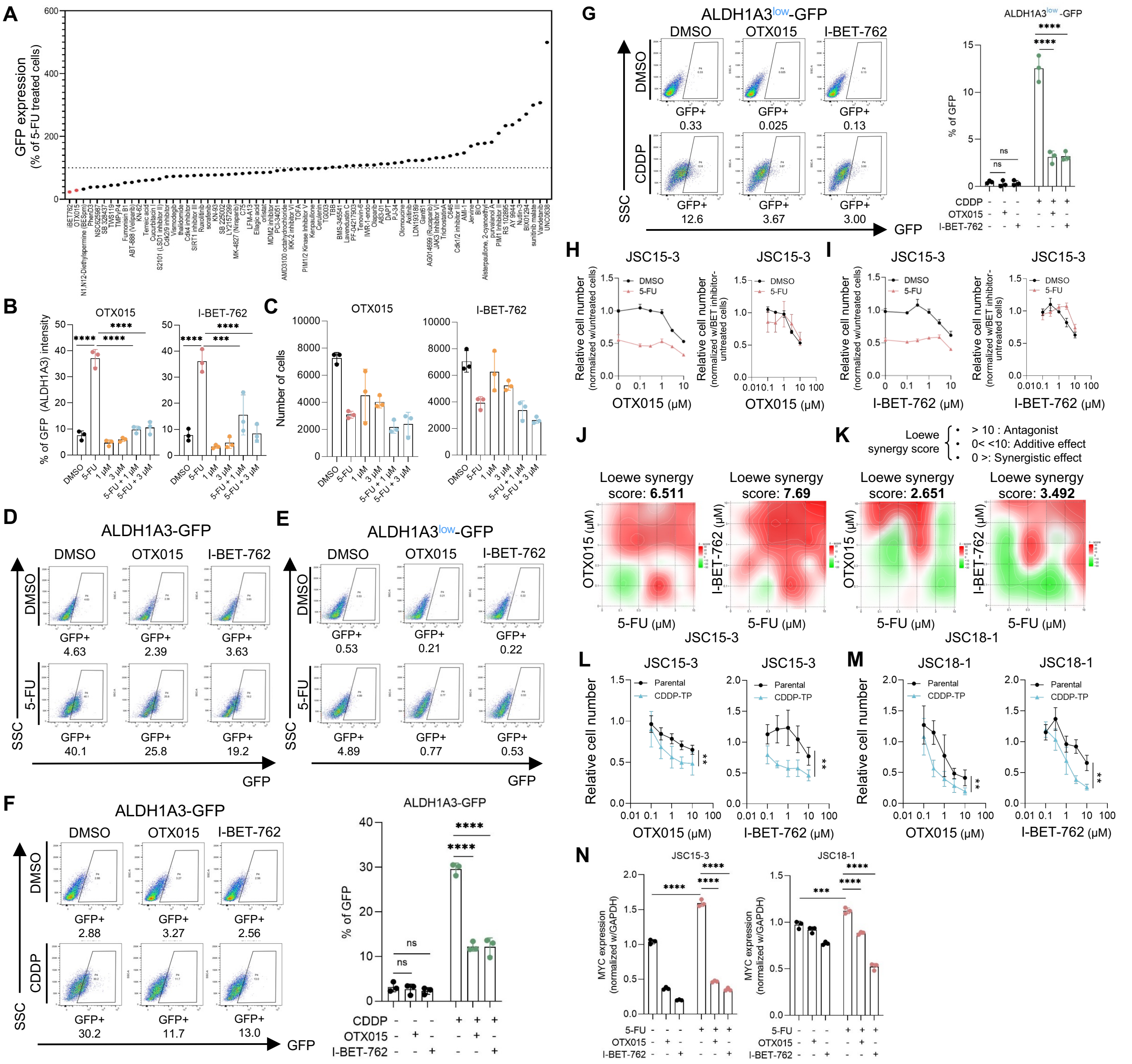

Supplementary Fig. S4 BET inhibitors suppress ALDH1A3 upregulation and preferentially inhibit DTP cell growth

A. Screening for drugs that inhibited 5-FU-induced ALDH1A3-GFP expression. Cells were treated with 3 µM 5-FU in the presence or absence of 3 or 4 µM test compound for 5 days. **B, C.** Effects of BET inhibitors on GFP expression (B) and cell number (C) in 5-FU-teated cells. ALDH1A3-GFP cells were treated with 3 µM 5-FU in the presence or absence of 1-3 µM OTX015 or I-BET-762 for 5 days (left: OTX015, right: I-BET-762). **D, E.** Raw data of FACS analysis of ALDH1A3-GFP (D) and ALDH1A3^{low}-GFP (E) cells in Fig. 4B. Cells were treated with 3 µM 5-FU in the presence or absence of 1 µM OTX015 or 3 µM I-BET-762 for 5 days. **F, G.** FACS analysis of ALDH1A3-GFP (F) and ALDH1A3^{low}-GFP (G) JSC15-3 cells. Cells were treated with 10 µM (ALDH1A3-GFP cells) or 1 µM (ALDH1A3 ^{low}-GFP cells) CDDP in the presence or absence of 1 µM OTX015 or 3 µM I-BET-762 for 5 days and subjected to flow cytometry. Experiments were performed with three technical replicates and were repeated at least three times. ****p<0.0001, ns: not significant, one-way ANOVA. **H, I.** Combinational effects of OTX015 or I-BET-762 and 5-FU (3 µM). Cells were treated with the agents for 5 days, and the cell number was measured. Left: relative cell numbers normalized with the numbers of untreated cells, which visualized the extent of additive effect of two drugs. Right: relative cell numbers normalized with cell numbers without BET inhibitors treatment. **J, K.** Synergistic effects of 5-FU and BET inhibitors on JSC15-3 (J) and JSC18-1 (K) cell growth. Cells were treated with the indicated concentrations of 5-FU with OTX015 (left) or I-BET-762 (right) for 6 days. Synergy scores were calculated using the Loewe model in SynergyFinder2.0 (https://synergyfinder.fimm.fi/synergy/synfin_docs/). The summarized Loewe synergy scores can be interpreted as indicated upper right in K. **L, M.** Effects of BET inhibitors on the parental and CDDP-tolerant persister (CDDP-TP) JSC15-3 (L) and JSC18-1 (M) cell growth. CDDP-TP cells were prepared by treating the respective parental cells with 10 µM (JSC15-3) or 1 µM (JSC18-1) CDDP for 5 days. Then, the cells were reseeded and treated with the indicated concentrations of OTX015 (left) and I-BET-762 (right) for 6 days. Experiments were performed with six technical replicates and were repeated at least three times. **p<0.01, one-tailed Mann–Whitney rank-sum test. **N.** Effects of BET inhibitors on 5-FU-induced mRNA expression of MYC in JSC15-3 (left) and JSC18-1 (right) cells. Cells were treated with 3 µM 5-FU in the presence or absence of 1 µM OTX015 or 3 µM I-BET-762 for 5 days. Then, RNAs were prepared and subjected to RT-qPCR.
