# Supplementary figures and images for: Pharmacologic Targeting of Histone H3K27 Acetylation/BRD4-dependent Induction of ALDH1A3 for Early-phase Drug Tolerance of Gastric Cancer
Source: Cancer Res Commun. 2024 May 20;4(5):1307–20. doi: 10.1158/2767-9764.CRC-23-0639 (PMC11104289; doi:10.1158/2767-9764.CRC-23-0639)

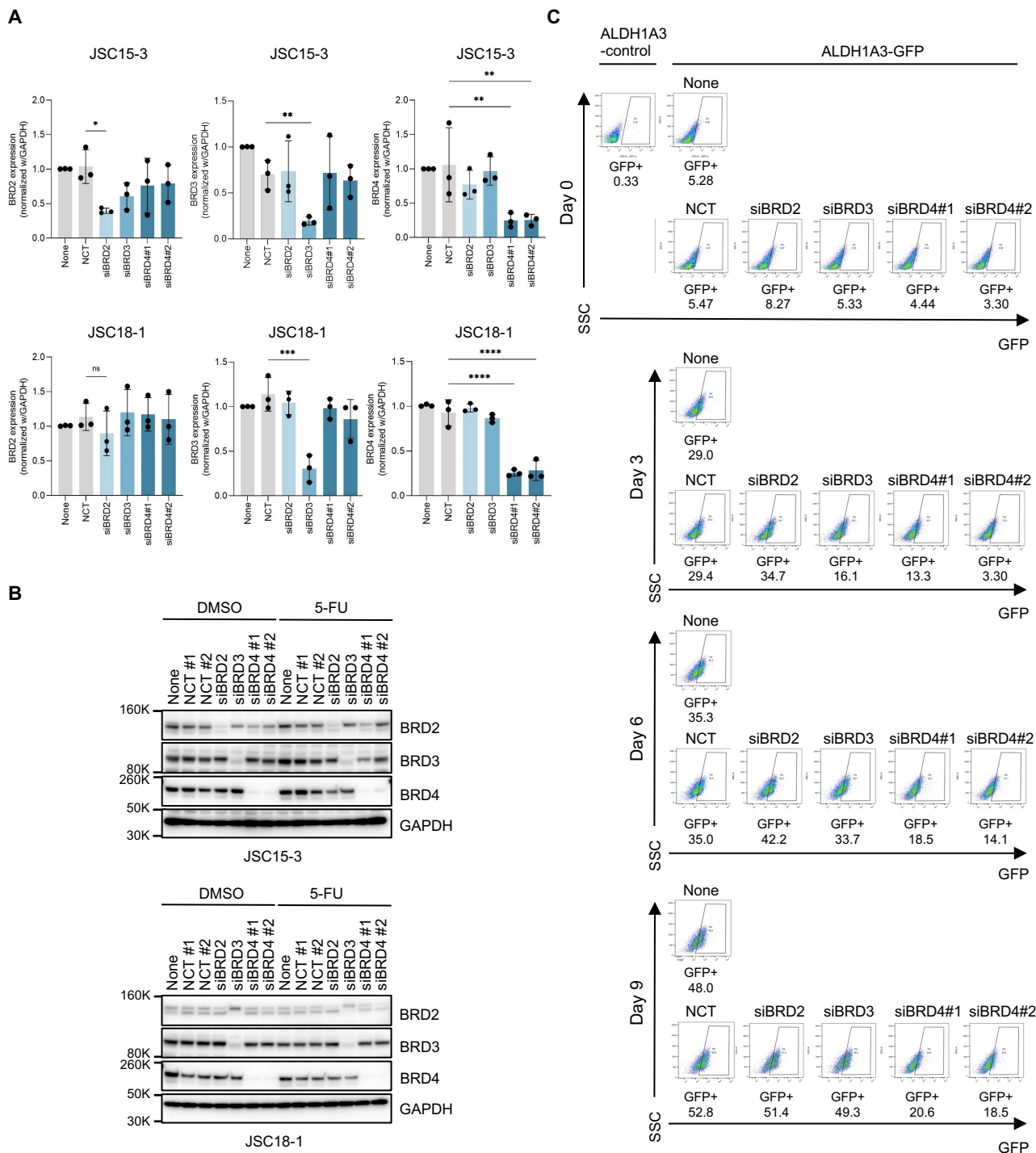

Supplement: Supplementary Figure S5 — BRD4 knockdown suppresses 5-FU-induced ALDH1A3 upregulation. [file crc-23-0639-s09.pdf]
