# Supplementary material for: Pharmacologic Targeting of Histone H3K27 Acetylation/BRD4-dependent Induction of ALDH1A3 for Early-phase Drug Tolerance of Gastric Cancer
Source: Cancer Res Commun. 2024 May 20;4(5):1307–20. doi: 10.1158/2767-9764.CRC-23-0639 (PMC11104289; doi:10.1158/2767-9764.CRC-23-0639)
Supplement: Supplementary Figure S6 — Estimation of ALDH1A3 gene amplification and the levels of biomarkers in 5-FU treated xenograft tumors [file crc-23-0639-s10.pdf]

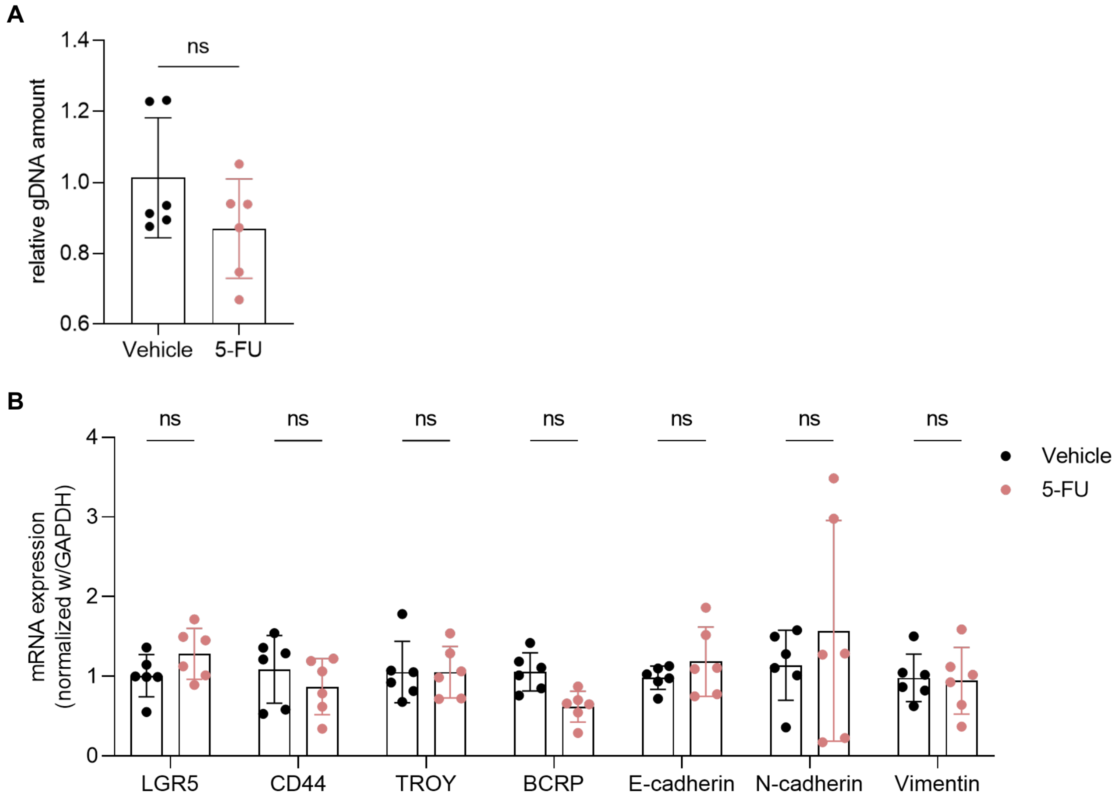

Supplementary Fig. S6. Estimation of *ALDH1A3* gene amplification and the levels of biomarkers in 5-FU treated xenograft tumors.

A. Evaluation of *ALDH1A3* gene amplification in 5-FU-treated xenograft tumors. Xenografted mice were treated as in Fig. 6A. gDNA was prepared and the levels of *ALDH1A3* gene copy were evaluated by qPCR. **B.** mRNA expression of cancer stem markers (CD44, Lgr5), stem cell marker (TROY), drug-resistant marker (BCRP), and EMT markers (E-cadherin, N-cadherin, Vimentin) in 5-FU-treated xenograft tumors (N = 6). Statistical significance was evaluated by t-test. ns: not significant.
